# Hatchery type influences the gill microbiome of Atlantic farmed salmon (*Salmo salar*) after transfer to sea

**DOI:** 10.1186/s42523-024-00347-y

**Published:** 2024-11-08

**Authors:** Kelly J. Stewart, Annette S. Boerlage, William Barr, Umer Z. Ijaz, Cindy J. Smith

**Affiliations:** 1 Infrastructure and Environment, James Watt School of Engineering, University of Glasgow, Glasgow, Scotland, UK; 2 Centre for Epidemiology and Planetary Health (CEPH), SRUC School of Veterinary Medicine, Scotland’s Rural College (SRUC), Inverness, UK

**Keywords:** Atlantic farmed salmon, Hatchery, Seawater, 16S rRNA, Gill microbiome

## Abstract

**Background:**

Salmon aquaculture involves freshwater and seawater phases. Recently there has been an increase in multifactorial gill health challenges during the seawater phase which has led to an urgent need to understand the gill microbiome. There is a lack of understanding on what drives the composition of the gill microbiome, and the influence the freshwater stage has on its long-term composition. We characterise the gill microbiome from seven cohorts of Atlantic salmon raised in six different freshwater operational systems—recirculating aquaculture system (RAS), flowthrough (FT) and loch-based system, prior to and after transfer to seven seawater farms, over two different input seasons, S0 (2018) and S1 (2019).

**Results:**

Using the V1-V2 region of the 16S rRNA gene, we produced amplicon libraries absent of host contamination. We showed that hatchery system influenced the gill microbiome (PERMAOVA R^2^ = 0.226, *p* < 0.001). Loch and FT systems were more similar to each other than the three RAS systems, which clustered together. On transfer to sea, the gill microbiomes of all fish changed and became more similar irrespective of the initial hatchery system, seawater farm location or season of input. Even though the gill microbiome among seawater farm locations were different between locations (PERMAOVA R^2^ = 0.528, *p* < 0.001), a clustering of the gill microbiomes by hatchery system of origin was still observed 7–25 days after transfer (PERMAOVA R = 0.164, *p* < 0.001). Core microbiomes at genera level were observed among all fish in addition to freshwater only, and seawater only. At ASV level core microbiomes were observed among FT and loch freshwater systems only and among all seawater salmon. The gill microbiome and surrounding water at each hatchery had more shared ASVs than seawater farms.

**Conclusion:**

We showed hatchery system, loch, FT or RAS, significantly impacted the gill microbiome. On transfer to sea, the microbiomes changed and became more similar. After transfer, the individual sites to which the fish were transferred has a significant influence on microbiome composition, but interesting some clustering by hatchery system remained. Future gill disease mitigation methods that target enhancing the gill microbiome may be most effective in the freshwater stage, as there were more shared ASVs between water and gill at hatchery, compared to at sea.

**Supplementary Information:**

The online version contains supplementary material available at 10.1186/s42523-024-00347-y.

## Background

Atlantic salmon, *Salmo salar* (Linnaeus, 1758), is one of the most valuable and widely farmed species globally, accounting for a 4.5% share of total farmed finfish (FAO, 2022). The production cycle of farmed Atlantic salmon begins with hatched fish reared in freshwater for ~ 10–16 months, after which fish undergo smoltification, followed by transfer to seawater to grow to harvest size. During the freshwater growth phase, salmon can be reared in freshwater lochs (Scotland), flow-through systems (FT) or recirculation systems (RAS). Most freshwater production takes place in RAS and FT systems [38]. Recirculating aquaculture systems are efficient, highly productive intensive farming systems that are not affected by variation in the environment because the water parameters are completely controlled and up to 99% of water is treated through biofiltration systems [13, 14, 30, 34]. Flowthrough (FT) systems do not provide the same degree of control as RAS, but more than open net pens in lochs as water flow and quality can be controlled, but they rely on nearby water sources to flow through the facility [4].

Like other mucosal sites, the gill mucosa contains a wide variety of biologically active compounds and immune cells that act to protect the fish from pathogenic invasion [16, 50]. In addition to intrinsic factors providing pathogen defence, microbial communities present in the gill mucosa may contribute to protection [3, 39]. Several mechanisms have been suggested for this including niche exclusion of pathogens and essential resource competition to suppress the growth of disease-causing microbes [16, 50]. Moreover, shifts in microbial community composition can result in disease due to dysbiosis and interactions with the host’s immune system [19, 53].

In recent years, growing attention has been directed towards external mucosal sites such as the gills [9, 30]. These studies have demonstrated that the gill microbiome in farmed salmon is dynamic and diverse [5, 12, 30] and changes over time, disease treatments [43], sampling methodology and rearing environment [42]. Furthermore, as our understanding of the gill microbiome expands, so does the aim to investigate the potential role of bacteria in gill health [5, 7, 18], given that a number of infectious organisms including bacteria have been linked to gill disease [6, 23]. Understanding gill health is therefore very important, and one of the ways to understand gill health better is understanding its microbiome.

In freshwater, the type of hatchery system has been shown to influence salmon gill microbiomes [30, 34, 42]. In a comparison of two RAS and one FT hatchery, the gill microbiomes in the RAS systems where more similar to one another than to the FT reared salmon [34]. Furthermore, the gill microbiomes in RAS reared fish appeared to be influenced by the surrounding water microbiome [30]. Despite difference among sites, core microbiomes among the gill microbiome in freshwater have been observed [30]. The transfer of fish between freshwater rearing units [35], and from freshwater to seawater [17, 28, 30, 31, 44] has been shown to alter the structure and composition of the skin and gut microbiome of Atlantic salmon [28]. As the transfer to sea drastically alters the community composition in the gills [30] and other mucosal organs [28, 31, 44, 49], it is unclear if differences in the microbiome at hatchery affect the microbiome composition in salt water, and whether season of transfer affects this. Understanding this, would aid inform the factors that influence the microbiome in seawater salmon such as how rapidly the overhaul in communities occurs at sea and might be an important step towards using knowledge to enhance gill health.

While an evaluation of gill microbiomes linked to fish genetics has also been investigated [8], the majority of gill microbiome studies have been carried out on a single population [17, 28, 31, 35, 44] from a limited number of sites, seasons and fish. Due to the large amount of variability encountered in the dynamic gills, snap-shot studies may not inform the full complexities of factors at play.

To address this, we compared the mucosal gill microbiomes of seven different cohorts of Atlantic Salmon, reared at six hatcheries of varying water systems—three RAS and two Lochs and a Flowthrough, before and after transfer to seven different seawater farms in Scotland over different input seasons Autumn 2018 and Spring 2019. We compared the gill microbiomes between three hatchery types and the changes that occur on transfer to sea. To further explore the influence of hatchery or seawater farm on the gill microbiome, the surrounding water for the Spring 2019 hatchery and seawater samples was examined.

## Methods

### Experimental design

**Fig. 1:**
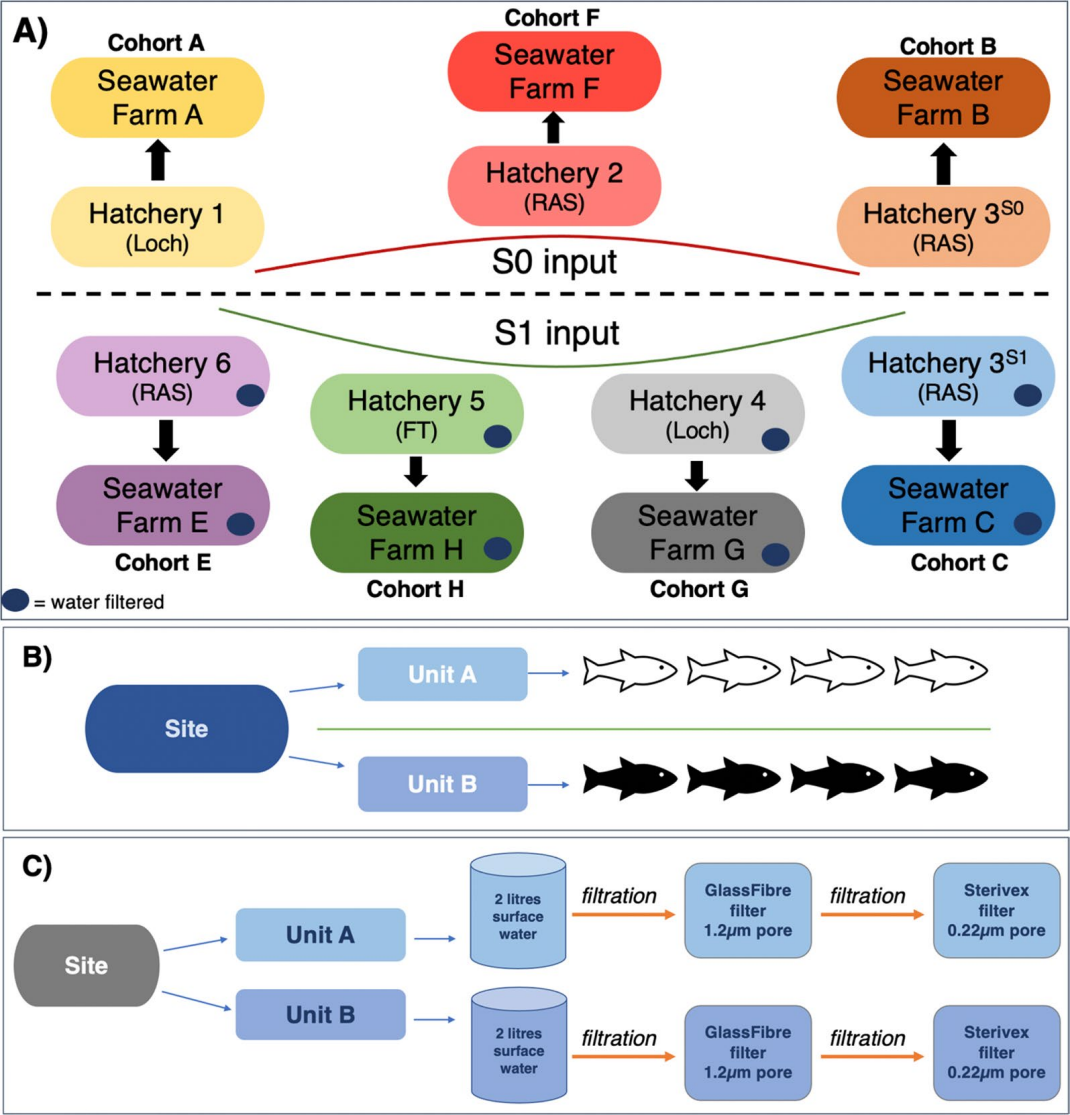
Experimental design overview. Information was pseudonymised for the experiment, with each hatchery identified by a number and each farm by a letter. (**A**) Gill microbiomes were sampled at 13 sites: 6 freshwater hatcheries and 7 seawater farms, across Scotland from S0 (Autumn 2018) and S1 (Spring 2019) inputs. Each of the seven cohorts were sampled at their hatchery then their corresponding seawater farm following transfer. Dark blue circles seen for all S1 sites indicates that surface unit water was sampled and filtered. Hatcheries 3^S0^ and 3^S1^ were the same site, sampled once for each seasonal input. (**B**) At each sampling point, four fish were sampled from Unit A and four fish from Unit B (n = 8 fish per site). (**C**) 2L of water was taken from the surface of each unit. The water was filtered through first through a 1.2 μm pore glass fibre filter (particle attached microbiome) and then a 0.22µm pore Sterivex filter (pelagic microbiome)

Seven cohorts of farmed Atlantic salmon were sampled twice, first at freshwater hatchery then after transfer at their seawater farm across 13 sites; 6 hatcheries (2 Loch, 3 RAS, 1FT) and 7 seawater farms (Fig. 1). Three cohorts were sampled within their year of hatching, termed “S0 inputs” while the remaining four were sampled in the year following their hatching, termed “S1 inputs” (Fig. 1A). S0 fish represent off-season smolts and typically transfer to sea in the autumn, while S1 fish represent one-season smolts and are typically transferred to sea in the spring according to standard industry practices in Scotland. At each site, 4 salmon from each of two units, representing cage, tank or sea pen depending on location, termed “A” and “B” were caught and sampled, producing n = 8 salmon per site (Fig. 1B). Hatchery salmon were sampled between 6 and 30 days before transfer, then 7–25 days after transfer once at sea, allowing salmon to acclimatise to their new environment for at least a week before sampling (Table 1). For each of the eight S1 input sites (four freshwater, four seawater), unit surface water was sampled (Fig. 1C). Approximate weights of fish in each unit for all cohorts at time of sea stocking is shown in grams in Table 1.

**Table 1:** Freshwater and seawater sites, date of sampling, sea stocking size and time at sea

Cohort	Freshwater hatchery—water system	Freshwater hatchery Sample date	Date of input to Sea	Sea stocking weight (avg. grams)*	Farm sample date	Days at sea before sampling*
A	H1—Loch	2/10/2018	01/11/2018	105/105	08/11/18	7/7
F	H2—RAS	29/10/2018	16/11/2018	120/120	04/12/18	18/16
B	H3^so^- RAS	25/10/2018	31/10/2018	83/83	12/11/18	12/12
C	H3^s1^- RAS	8/03/2019	(A)29/03–2019 & (B) 02/04/2019	124/105	17/04/19	19/15
G	H4—Loch	4/01/2019	10/01/2019	75/79	22/01/19	12/12
H	H5- Flowthrough	13/03/2019	08/04/2019	67/57	03/05/19	25/25
E	H6—RAS	29/04/2019	(A)18/05/2019 & (B) 22/05/2019	122/87	06/06/19	19/15

*Individual results for Rearing Unit A/Rearing Unit B (A/B) are shown, separated by /

### Gill mucosal and water microbiome sampling

Fish were randomly selected and caught using dip or cork nets and sedated using tricaine methanesulfonate, Pharmaq, 1000mg/g (MS- 222), according to UKs Veterinary Medicines Directorate. Samples were taken by trained site staff or fish health representatives from each of the different companies involved in the project. All animal procedures were approved by the consortiums lead institute, Scotland’s Rural College (SRUC) Animal Ethics Committee. Hemibranches 2, 5, 6 and 7 of the right gill of each fish were swabbed using eNat™ (COPAN Diagnostics Inc.) swabs, brushing up and down to fully gather the gill mucus. Swabs were immediately frozen at − 20C until return to the laboratory for storage at -80 C until processing. For S1 inputs, 2L of surface water was collected from each rearing unit at sampled sites and filtered through a 1.2μm GF/C glass fibre filter (Whatman, UK) to recover microbial aggregates and onto a 0.22μm Sterivex (St) filter (Merck, UK) to recover pelagic bacterial cells, creating two water samples per unit (Fig. 1C). Filters were sealed in sterile containers and frozen at − 20°C until transfer to the laboratory. Samples were stored at − 80°C until processing.

### DNA extraction and 16S rRNA gene amplicon library preparation

DNA extraction from eNat™ swabs (mucosal gill microbiomes) were optimised to ensure collection of all DNA from potentially low biomass samples (Supplementary Fig. 1). Briefly, swabs were thawed on ice and gently sonicated for 2 min at 40kHz to remove bacterial cells after which the supernatant (SA) was transferred to a sterile 1.5mL Eppendorf tube. The remaining swab head was washed with 800μL of Phosphate Buffered Saline (PBS), vortexed briefly to detach any remaining cells, and the supernatant (SB) was transferred to a sterile 1.5mL Eppendorf tube, resulting in two tubes of supernatant, SA and SB. Next, the swab was cut from its stem, centrifuged at max speed for 10 s to collect the remaining supernatant, adding this to the previous S1 Eppendorf. SA and SB were then centrifuged at 12,000g for 20 min at 4°C to pellet bacterial cells. The supernatant of these tubes was removed and transferred to a sterile 2.0mL tube while not disturbing the pelleted cells, creating, SC and SD. In case bacterial cells were lysed during the thawing and sonication steps, SC and SD were ethanol precipitated with 1/10 sodium acetate solution (3M, pH 5.2, Thermo Fisher Science, UK) and 0.6–0.7 total sample volume of isopropanol (molecular biology grade, Sigma Aldrich, UK) according to Green and Sambrook 2016. DNA was precipitated for 30 min, followed by centrifugation at 14,000g for 30 min, followed by two 70% ethanol washes. The DNA pellets (DNA 1 and 2) were air dried and re-suspended in 20μL of DNase/RNase free water (Thermo Fisher Science, UK).

The two cell pellets were each resuspended in 90μL of lysis buffer (20mg/mL lysozyme; 20mM Tris.HCL, pH 8.0; 2mM EDTA; 1.2% Triton), combined (180μL total), and incubated at 37°C for 30 min; after which the QIAamp DNA Mini kit (Qiagen, UK) kit was used according to manufacturer’s instructions with a final elution of DNA (DNA 3) in 100μL elution buffer.

DNA 1–3 were combined and dehydrated using the Effendorf Concentrator plus (UK) by running the V-AQ mode at 45°C until pellets were fully dehydrated, and finally DNA was resuspended in 30μL of DNase free water. DNA extraction controls were completed using sterile blank eNAT swabs for each DNA extraction kit used (n = 3) as detailed above.

For water, each filter was defrosted on ice, then transferred to a sterile 2.0mL tube with 180μL of lysis buffer (20mg/mL lysozyme; 20mM Tris.HCL, pH 8.0; 2mM EDTA; 1.2% Triton), incubated for 30 min at 37°C, and processed using the QIAamp DNA Mini kit (Qiagen, UK) as detailed above.

### 16S rRNA amplicon library preparation for Illumina MiSeq

Dual indexed 16S rRNA gene primers F27 (5' AGAGTTTGATCMTGGCTCAG 3') (Lane et al., 1991) and R338 (5' GCTGCCTCCCGTAGGAGT 3') [1] adapted from Fadrosh et al. (2014) (Supplementary Table 1) targeting the V1-V2 region were selected due to their low alignment score to the Atlantic salmon 18S rRNA (F27 65% similarity; F338 72% similarity, Supplementary Table 2), in order to minimise host contamination in the amplicon library.

PCR was performed using HotstarTaq PCR (Qiagen, UK) in 25μL reaction volumes, using 1μL of template DNA in each reaction. PCR conditions were—95°C for 15 min followed by 35 cycles of 95°C for 1 min, 58°C for 30 s, 72°C for 30 s, and a final extension of 72°C for 10 min. Amplicons size, 490bp, was confirmed on 1% agarose gel. The products were cleaned with AMPure XP (Beckman Coulter, Agnecourt, UK) magnetic beads and quantified using the Qubit 1X dsDNA HS assay kit (Thermo Fisher Scientific). The 16S rRNA gene was amplified from 112 gill samples, 32 water samples, 3 negative controls, 3 even mock community samples (MSA-1000; ATCC, UK) and 3 uneven mock community samples (MSA-1001; ATCC, UK). Mock and negative controls were added for quality control of final sequencing results. The final amplicon pool was created to 34.06nM concentration, containing 20ng of each individual barcoded amplicon sample, was added to create the sequencing library. The pooled library was assessed using the Agilent 2100 Bioanalyser, Agilent DNA 1000 assay kit (Agilent, UK), and submitted to the Earlham Institute (Norwick, UK) for Illumina MiSeq (250PE) sequencing (one v2 flow cell lane, 20% PhiX spike). Sequences are available under accession number PRJEB65363, European Nucleotide Archive.

### Bioinformatics

Raw sequence data was custom demultiplexed by the Earlham Institute using the dual indexes to identify sequences specific to each sample. The Qiime2 workflow (V2019.7) and DADA2 pipeline [11] (https://github.com/umerijaz/tutorials/blob/master/qiime2_tutorial.md) was used to create ASVs. Briefly, reads were trimmed to remove indexes, bp spacers and primers using the cutadept package then trimmed to remove sections with a quality score below 30 (forward reads trimmed at 215bp, reverse reads trimmed at 200bp). The DADA2 pipeline [11] then dereplicated the reads, and chimeras were identified and removed using the isBimeraDenovo() function, and the prepared merged reader were classified into ASVs. The SILVA SSU v138 database was used to classify the ASVs, and Qiime2 was used to generate a rooted phylogenetic tree. Abundance information was combined with taxonomy in a BIOM file and all further statistical analysis was carried out in R v4.0.0 and R studio V1.3.959. Before downward analysis, the DeconSeq tool (v0.4.3; [45]) was used to identify any ASVs that matched to the reference *Salmo salar* 18S rRNA reference gene (Accession; FJ710886 from ENA 2019). The decontam package for R was used to identify and remove contaminants using negative controls (v1.10.0; [15]). Any samples, out with the negative controls, with < 5000 reads were removed from the study. Furthermore, ASVs that were singletons, unassigned, assigned as chloroplast or mitochondria and non-classified at phylum level were removed prior to analysis.

#### Statistical analysis

Rarefaction curves for quality control before analysis were created using R packages phyloseq (v1.46.0) McMurdie and Holmes, 2013) and vegan (v2.5-7; [40]) in relation to ASV richness. Mock community sequence data was assessed against expected proportions and abundance results to identify possible bias. Alpha and beta diversity was assessed in R using the Vegan package (v2.5-7; [40]). Alpha diversity measurements: Richness (rarefied) (no. of ASV’s in each sample that were rarefied to minimum library size) and Shannon entropy (logarithmic calculation of richness and evenness) were used to assess diversity within each individual sample. Pair-wise analysis of variance (ANOVA) testing was preformed between all sites, with significant p-values between two groups, with *p* < 0.05 (*), *p* < 0.01 (**), *p* < 0.001 (***). To assess differences between microbiomes, beta diversity measurements: Bray–Curtis distance (relative abundance) and Unweighted Unifrac (phylogeny) were plotted on PCoA plots with phyloseq (v1.46.0; McMurdie and Holmes, 2013). Permutational multivariate analysis of variance (PERMANOVA) was used to find sources for microbiome differences between groups using explanatory variables by employing Vegan’s adonis() function and incorporating different dissimilarity indices. This used pseudo-F ratios to determine the sources of variation, with R^2^ values (if significant) explaining the % variable by the chosen factors, with p values; *p* < 0.05(*), *p* < 0.01(**), *p* < 0.001(***). Samples were grouped by site, with ellipses drawn to represent the 95% confidence interval of the standard error of the group. Beta dispersion was used to analyse the multivariant homogeneity of the dispersion between units at each site using betadisper() in Vegan (v2.5-7; [40]). An ANOVA was used to compare centroids of each unit to one another to determine whether differences were significant. “Core microbiomes” groups of fish were calculated using the core microbiome package in R (v1.12.0; [26]) (https://microbiome.github.io/tutorials/Core.html) in terms of relative abundance. Core microbiomes were created using a minimal prevalence threshold, meaning any listed ASVs were present in 85% of gill microbiome of the target groups samples. Water, attached and pelagic fractions per sample were combined using a 50% core microbiome function, meaning that an ASV had to be present in one of the two samples. ASVs below an abundance of 0.1% were removed before creating each core microbiome.

## Results

### The number of bacterial taxa and the community structure of the gill microbiome changed on transfer to sea

**Fig. 2:**
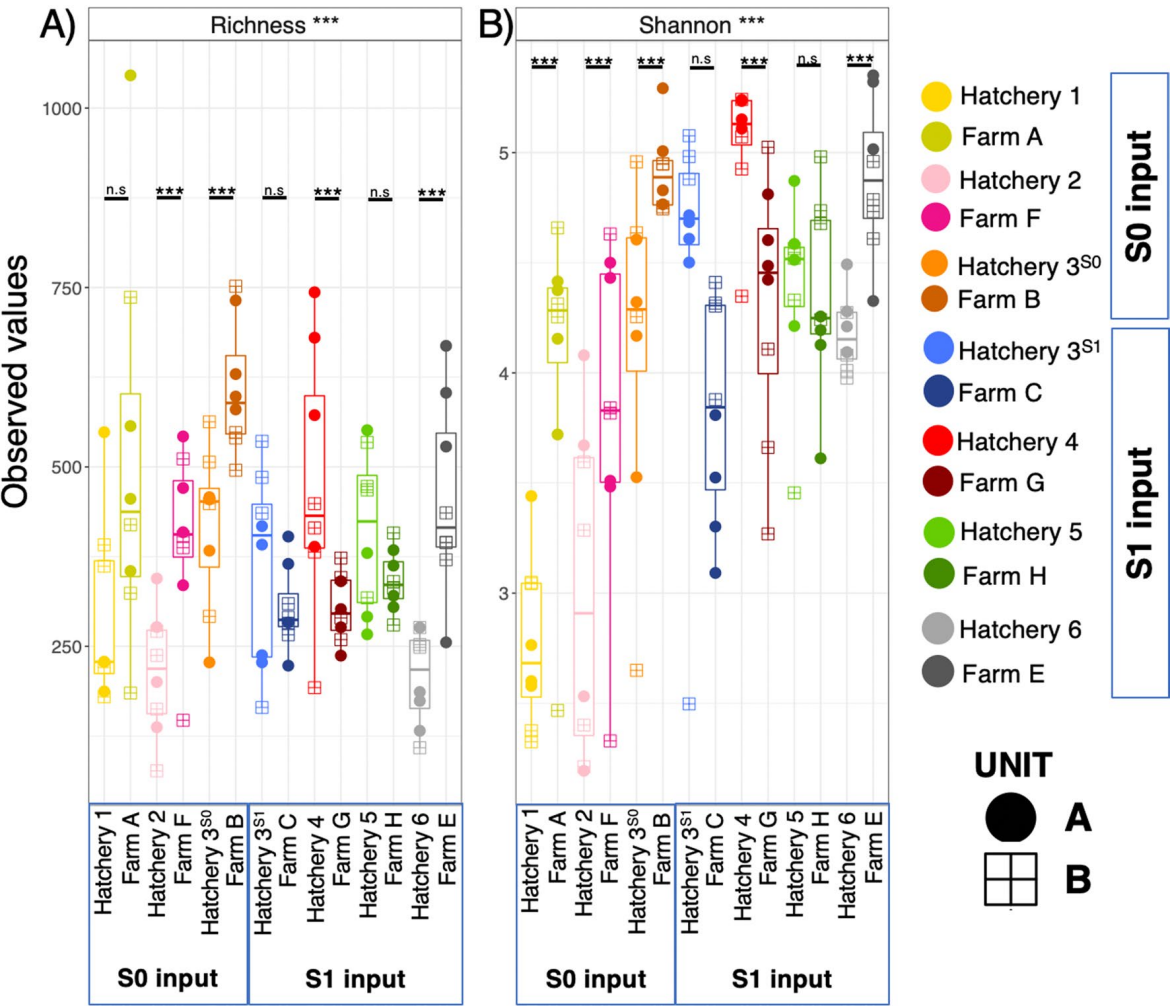
Alpha diversity of mucosal gill microbiomes of freshwater and seawater farmed Atlantic salmon. (**A**) Species richness (rarefied) (**B**) Shannon entropy. Grouped by site, with units A and B represented by different icons. Pairwise ANOVA p-values are hand drawn between each cohort, shown as *p* < 0.05*, *p* < 0.01**, *p* < 0.001*** and n.s = non-significant p value of > 0.05

The gill microbiome of Atlantic salmon species richness ranged between 222 (± 33 SE) ASVs at Hatchery 2 to 609 (± 32 SE) ASVs at Farm B (Supplementary Table 3) . For three cohorts, species richness increased on transfer to sea (F, B and E, *p* > 0.001), one decreased (G, *p* > 0.001) and three showed no statistical change on transfer to sea (A, C and H) (Fig. 2). As such there was an overall trend of increasing species richness for S0 inputs from hatchery on transfer to sea, but no trend observed for S1 inputs (Fig. 2). The species evenness for all S0 cohorts increased on transfer to sea (*p* > 0.001, Fig. 2B). However again, no trend was observed for S1 input (Fig. 2), with two cohorts showing no change (C and H), one increasing (E) (*p* < 0.001) and one decreasing (G) (*p* < 0.001) in species evenness after transfer to sea (Fig. 2B).

### Hatchery and seawater gill microbiomes differed, with hatchery water system influencing microbiome composition

**Fig. 3:**
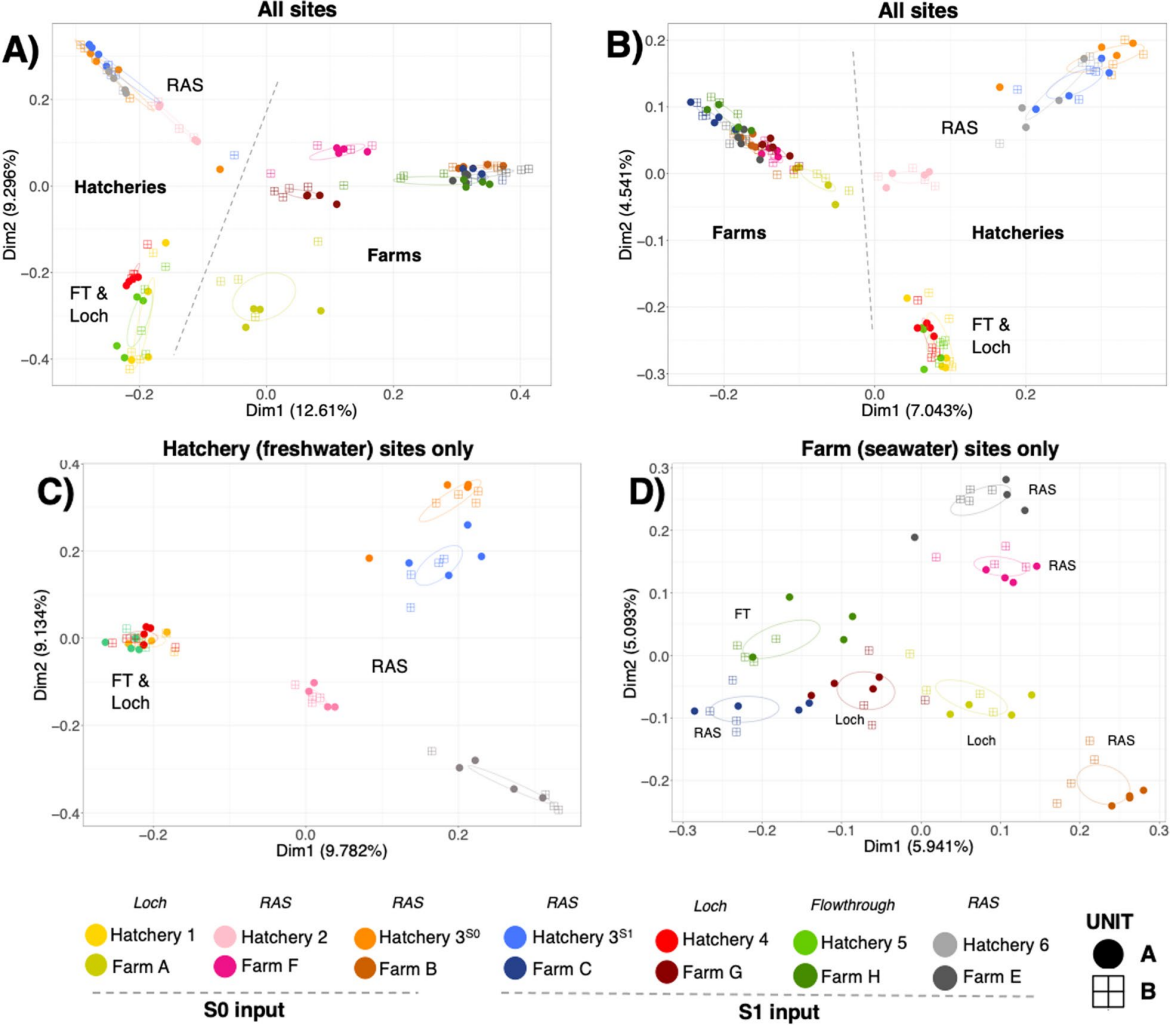
Beta diversity results of mucosal gill microbiomes of farmed Atlantic salmon. PCoA plots of Beta diversity measurements (**A**) Bray–Curtis dissimilarity of all Atlantic salmon (**B**) Unweighted Unifrac of all Atlantic salmon (**C**) Unweighted Unifrac of hatchery Atlantic salmon (**D**) Unweighted Unifrac of seawater farmed Atlantic salmon. Difference colours indicate difference sites, while ellipses represent 95% confidence interval of standard error of groups. Icons indicate salmon rearing unit. Hand drawn lines divides freshwater and seawater groups in A & B. FT = Flowthrough, RAS = Recirculating aquaculture system and Loch indicates water system of each hatchery and hatchery of origin for seawater sites

The gill microbiomes from hatchery and seawater clustered separately based on both the relative abundance of the taxa present (Bray Curtis, Fig. 3A) and the phylogenetic presence/absence of the taxa (Unweighted Unifrac, Fig. 3B), with the difference in water type (freshwater vs. seawater) significantly influencing microbiome composition in PERMANOVA analysis (Table 2, Bray Curtis—R^2^ 0.046, U.Unifrac—R^2^ 0.022, both *p* < 0.001). Site was a significant source of variance for the gill microbiomes, explaining > 50% of the variance in terms of relative abundance among all sites (R^2^ 0.580, *p* < 0.001), hatcheries only (R^2^ 0.539, *p* < 0.001) and seawater farms only (R = 0.528, *p* < 0.001) (Table 2), while phylogeny-based analysis explained 24–31.6% of differences (Table 2). The hatchery water system was a strong driver of the gill microbiome describing 22.6% and 13.1% of the variance for relative abundance and phylogeny of the gill microbiomes (R^2^ 0.226 & 0.131, *p* > 0.001, Table 2). The FT and two Loch hatchery systems highly similar to each other and the RAS systems clustered together to various degrees (Fig. 3B and 3C). Within the RAS systems, the gill microbiomes of fish samples from hatchery 3 at two different times, were the most similar (Fig. 3C). Once the smolts were put to sea, irrespective of the hatchery they came from, the gill microbiomes became more similar, but individual farm sites was still a driver of variance (R^2^ 0.528 & R^2^ 0.244, *p* > 0.001, Table 2 and Fig. 3D). Interestingly, although the gill microbiomes became more similar at sea, there was still a signal from the hatchery type of origin preserved (R^2^ 0.164 & R^2^ 0.070, *p* > 0.001, Table 2), and fish originating from the flow-through and Loch systems (cohorts A, G and H) clustered together while the RAS cohorts F and E were more similar to each other (Fig. 3B and 3C). However, the two cohorts (B & C) from the same hatchery (3) put to sea at two different input seasons to different sites were dissimilar. In addition, season of input (S0 verses S1), average input weight and days at sea has smaller, but nonetheless significant R^2^ values (Table 2).

**Table 2:** PERMANOVA for sources of variation between mucosal gill microbiomes for beta diversity measures Bray–Curtis and Unweighted Unifrac

Group	Sources of Variance	Bray–Curtis Dissimilarity R^2^ *p* < 0.001 (***)	Unweighted Unifrac R^2^ *p* < 0.001 (***)
All Atlantic salmon	Sites (n = 13)	0.580 (***)	0.316 (***)
Freshwater salmon	Hatchery sites (n = 6)	0.539 (***)	0.307 (***)
Seawater salmon	Farm sites (n = 7)	0.528 (***)	0.244 (***)
All Atlantic salmon n = 112	Season of Input (S0 vs S1)	0.098 (***)	0.057 (***)
	Water type (Freshwater vs. Seawater)	0.046 (***)	0.022 (***)
Freshwater hatchery salmon (n = 56)	Season of Input (S0 vs S1)	0.080 (***)	0.043 (***)
	Hatchery water system (Loch vs. RAS vs. FT)	0.226 (***)	0.131 (***)
Seawater farm salmon (n = 56)	Season of Input (S0 vs S1)	0.093 (***)	0.047 (***)
	Prev. hatchery water system (Loch vs. RAS vs. FT)	0.164 (***)	0.070 (***)
	Input weight (g)	0.078 (***)	0.036 (***)
	Days at Sea	0.096 (***)	0.038 (***)

R^2^ represents the variability explained between microbial communities. *p* < 0.1 (n.s), *p* < 0.05 (*), *p* < 0.01 (**), *p* < 0.001 (***). Each of the six titles in the Groups column represents a different PERMANOVA test

Duplicate rearing units were sampled at each hatchery and seawater farm to investigate whether there were differences in the gill microbiome of fish from different units at the same site. Gill microbiomes between units at hatchery were highly reproducible, with only cohort G, hatchery 4 (RAS), showing a statistical difference in the relative abundance (Bray Curtis R^2^ = 0.006, *p* < 0.01) and cohort H, hatchery 5 (FT) showing a statistical difference in the phylogeny of bacteria (U.Unifrac R^2^ = 0.009, *p* < 0.01) (Table 3). At sea, there was more variability observed between units at the same site, with difference in the relative abundances of bacteria on the gill of farm A, C and G (Table 3), in addition to difference in the phylogeny of bacteria on the gills at farm B and C (Table 3).

**Table 3:** Comparing the gill microbiomes of Units A and B at each site using beta dispersion

Cohort (input)	Site	Bray curtis beta dispersion *p* values	Unweighted unifrac beta dispersion *p* values
A (S0)	Hatchery 1	0.826	0.755
	Farm A	0.01*	0.115
F (S0)	Hatchery 2	0.144	0.472
	Farm F	0.123	0.237
B (S0)	Hatchery 3-S0	0.639	0.654
	Farm B	0.061	0.038*
C (S1)	Hatchery 3-S1	0.099	0.946
	Farm C	0.012*	0.009**
G (S1)	Hatchery 4	0.006**	0.417
	Farm G	0.044*	0.057
H (S1)	Hatchery 5	0.371	0.009**
	Farm H	0.239	0.782
E (S1)	Hatchery 6	0.078	0.141
	Farm E	0.689	0.53

Values shown represent the *p* values, with asterixis representing statistically significant results. *p* < 0.05 (*), *p* < 0.01 (**), *p* < 0.001 (***)

### The dominant taxa of the gill microbiome changed following transfer to seawater and varies by site

A total of 8829 ASVs were observed in the gills of freshwater hatchery fish and 11,726 ASVs on the gills of seawater farm fish. The ASVs represented a total of 47 Phylum, with the ten most abundant phylum of the mucosal gill microbiomes encompassing at least 92% or greater of the dominating phylum (Supplementary Fig. 2). The remaining 8% representing the non-listed 37 other phylum. Prominent phylum include Proteobacteria, present in > 50% on average per site expect hatchery 4 (47.7%) and 6 (35.3%), followed by *Bacteriodota (*range from 8 to 55.6% per site*)* and *Actinobacteriota (*range from 0.15 to 22.6% per site*)* (Supplementary Fig. 2).

**Fig. 4:**
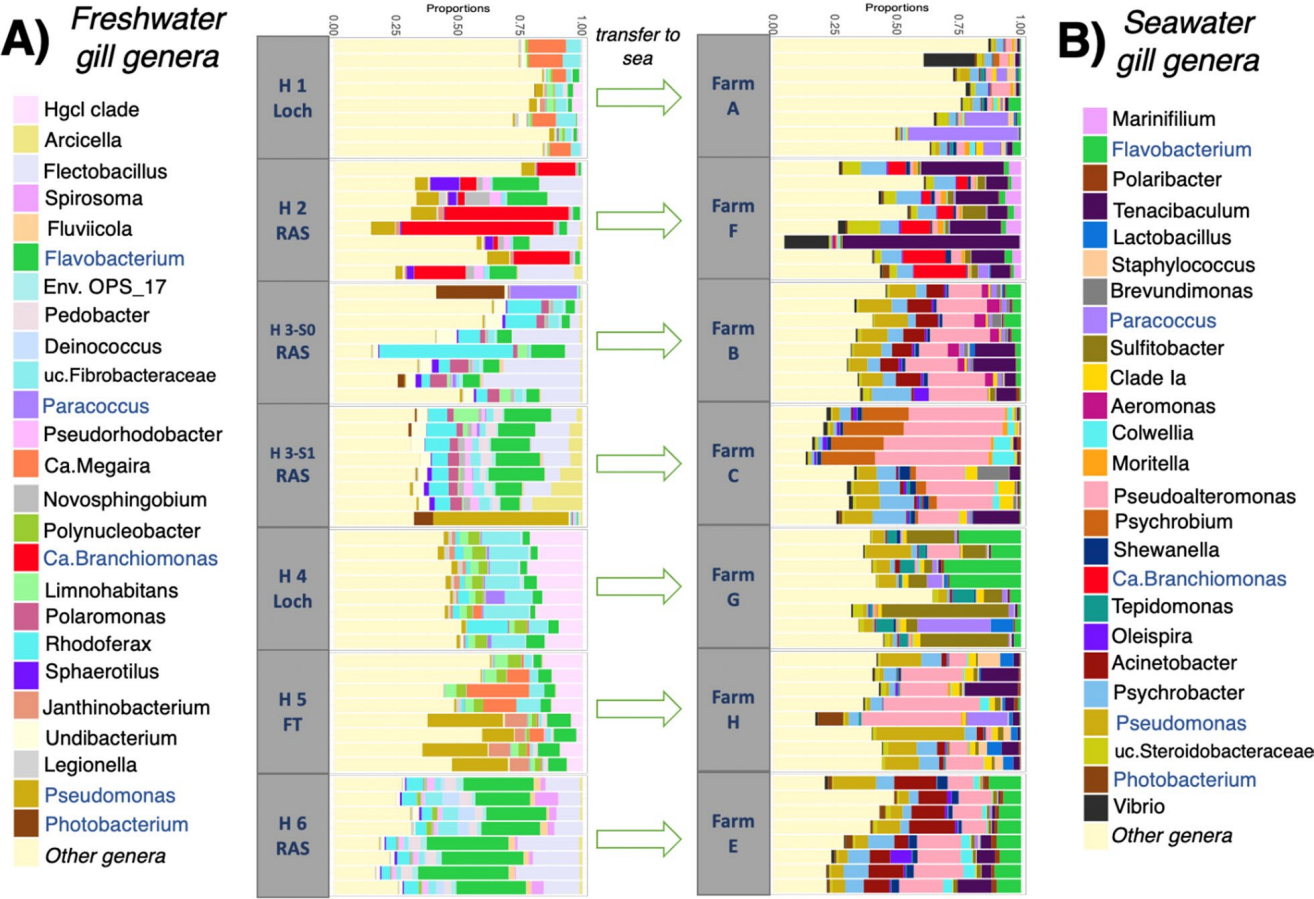
Most abundant genera of freshwater and seawater farmed Atlantic salmon. (**A**) Twenty-five most abundant genera of freshwater hatchery salmon (**B**) Twenty-five most abundant genera of seawater farm salmon. Each bar represents an individual salmon gill microbiome, plotting fish 5–8 from unit A, then fish 5–8 from unit B in series. Samples grouped by site, with each column representing an individual fish

At genus level, the relative abundances of the top twenty-five taxa for both freshwater hatchery and seawater gill microbiomes varied largely between sites and fish themselves (Fig. 4). In freshwater salmon, twenty-five taxa account for greater than 50% of the abundant taxa of the gills, with the exception of Hatchery 1 where the same taxa accounted for just 13.1–28.1% of present genera in the gills of each fish (Fig. 4A). In the gills of freshwater salmon, *Flavobacterium* was a constant presence (1.1–25.2%) on all 56 salmon, as was *Fibrobacteraceae* (0.86–13.4%) and *Pseudomonas* (0.5–11.8%). Hatchery water system influenced the top 25 taxa, with *hgcl_clade* found in hatcheries 1, 4 and 5 which employed either a FT or Loch based systems, at a relative abundances of 1.5%, 17.3% and 9.1% respectively. In comparison, for the RAS based systems (hatcheries 2, 3^S0^,3^S1^ and 6), *Flectobacillus* was present at high relative abundances between 9 and 15.8% (Fig. 4A).

A complete shift in the most abundant genera of the gills occurred after transfer to seawater farms (Fig. 4B). In seawater salmon, the top twenty-five taxa account for greater than 58% of the average abundant gill taxa, with the exception of Cohort A where they are much lower accounting for on average just 31.2% of detected genera in the gills (Fig. 4B). The genera *Pseudoalteromonas* and *Flavobacterium* were abundant at all farms, ranging in relative abundance from 1.7 to 31.7% and 0.26 to 13.5% respectively. *Candidatus Branchiomonas* was present in the top 25 taxa at 3 farms; H (3 salmon—0.1–0.3%), C (3 salmon—0.1–0.5%) and F (all salmon—0.8–21.3%). *Ca. Branchiomonas* was also observed in freshwater hatchery 2 (Cohort F) on all fish with a relative abundance between 1.9 and 61.1%. In total there are five genera from the top 25 taxa present in both freshwater hatcheries and seawater; *Flavobacterium*, *Paracoccus*, *Candidatus Branchiomonas*, *Pseudomonas* and *Photobacterium* (Fig. 4).

Beyond the top 25 taxa, the presence of shared genera between fish was explored to determine if there was a farmed Atlantic salmon “core” microbiome, with core defined as being present in at least 85% of all fish. Across all salmon (n = 112) at genera level, this consisted of *Flavobacterium*, *Pseudomonas,* and an uncultured *Fibrobacteraceae* (Supplementary Fig. 3A), while unsurprisingly, at ASV level there was no core microbiome detected among all salmon.

We next asked if a core microbiome was present among freshwater sites only (Supplementary Fig. 3B) and seawater sites only (Supplementary Fig. 3C). At genera level, a freshwater core microbiome of the gills was found among all sites composed of only two genera, *Flavobacterium*, and an uncultured *Fibrobacteraceae*, with no ASV level core detected.

At genera level, a seawater core microbiome (Supplementary Fig. 3C) was found among all sites composed of 10 genera; *Flavobacterium*, *Vibrio, Shewanella, Sulfitobacter, Clade_Ia*, uncultured *Steridobacteraceae, Tenacibaculum, Pseudomonas, Pseudoalteromonas* and *Psychrobacter*. Furthermore, a core microbiome of 2 ASVs was found among 85% or more of seawater salmon gills (Supplementary Fig. 3D); ASV_12 *Psychrobacter* and ASV_3 *Pseudoalteromonas* at relative abundances between 0.1 and 0.54% on the gills.

Given the variation seen between freshwater sites, we further explored “core” groupings by examining LOCH/FT freshwater sites only and RAS sites only, detecting core gill microbiomes for both groups (Supplementary Fig. 4). A total of 6 genera were shared between all RAS farmed freshwater salmon; *Arcicella, Novosphingobium, Undibacterium*, uncultured *Steridobacteraceae*, *Flectobacillus* and *Flavobacterium*, while no shared core was found at ASV level for the grouping (Supplementary Fig. 4A). Across loch and FT hatchery fish, 9 shared genera were found to compose a core microbiome; *Shingomonas, Sediminibacterium, Clade_III, Pseudomonas, Limnohabitans, Ploynucloebacter, Flavobacterium, Hgcl_clade* and uncultured *Fibrobacteraceae* (Supplementary Fig. 4B). Furthermore, 5 core ASVs were found upon further investigation (Supplementary Fig. 4C) across the loch/FT group, but at very low relative abundances of 0.1–0.3%. Comparison of the results only showed *Flavobacterium* as shared between both groupings (RAS vs. Loch/FT).

### Influence of surrounding water on the gill microbiome

**Fig. 5:**
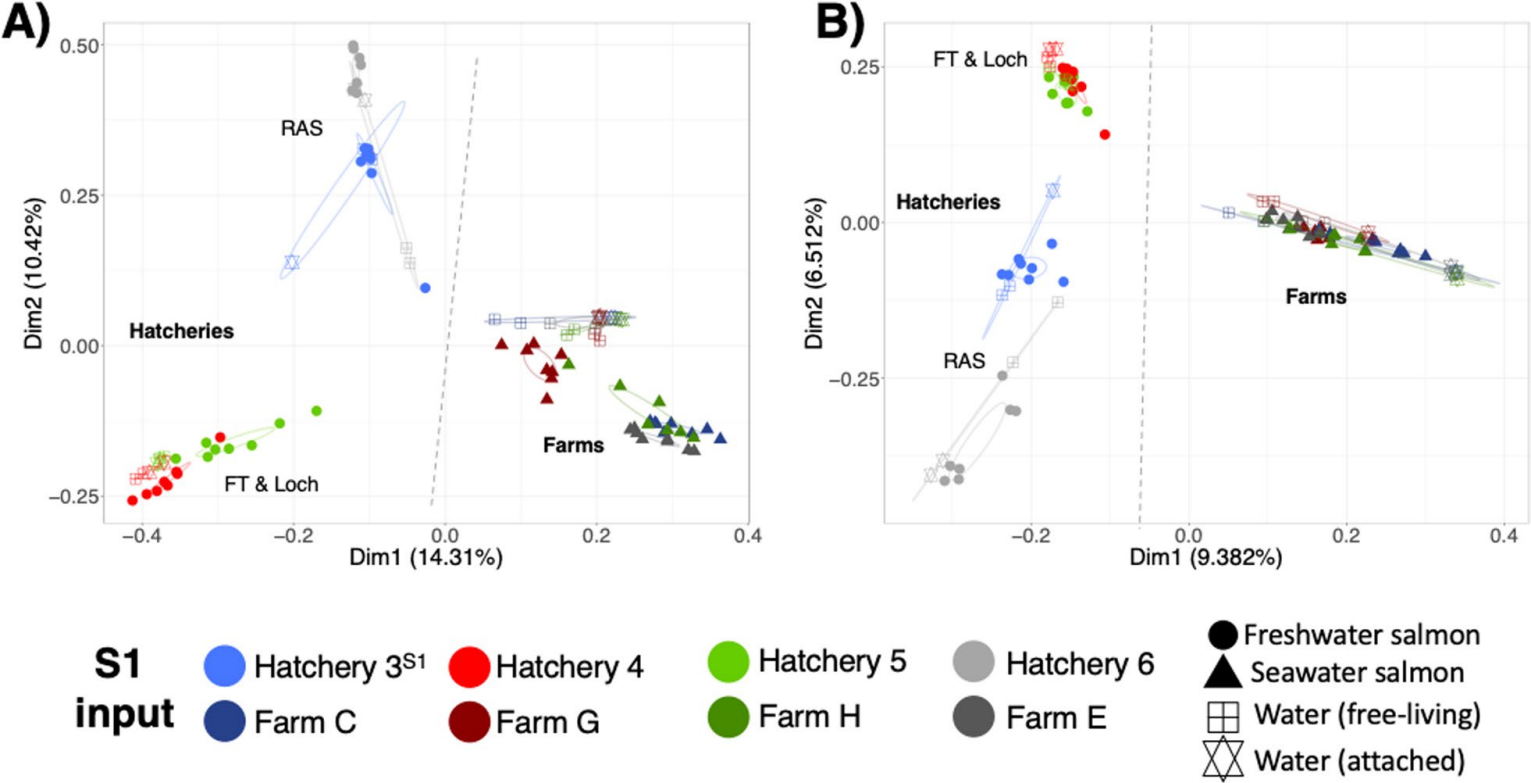
Beta diversity results of mucosal gill and water microbiomes of farmed Atlantic salmon from the S1 input. PCoA plots of Beta diversity measurements (**A**) Bray–Curtis dissimilarity (**B**) Unweighted Unifrac. Hand drawn lines divide freshwater hatchery and seawater farm samples. Each colours indicate different site, while ellipses represent 95% confidence interval of standard error of groups. FT = Flowthrough, RAS = Recirculating aquaculture system and Loch indicates water system of hatchery

To examine the influence of the surrounding water microbiome on the gills, water samples were taken from hatcheries and seawater cages for all S1 inputs. Water was prefiltered though a 1.2 μm pore glass fibre filter (particle attached microbiome) onto a 0.22μm pore Sterivex filter (pelagic microbiome) and DNA extracted and sequenced from both fractions. Within the hatcheries, the top 25 taxa of the water from the Loch and Flowthrough hatcheries were similar, while the two RAS systems were very different (Supplementary Fig. 5). In seawater, the top 25 taxa were more consistent and similar across the four sites. These findings were mirrored in the beta diversity plots of gill and surrounding water microbiomes (Fig. 5). In seawater, the water microbiome from different locations was highly similar and clustered closely with the gill microbiome, in particular for the Unweighted Unifrac analysis (Fig. 5B), indicating a high degree of phylogenetic similarity. For the freshwater hatcheries, the water and gill microbiomes from the RAS systems were more similar to each other, than to the FT and Loch systems, where both the gill and the hatchery water microbiomes were highly similar. Hatchery type and its water was a significant driver of differences (Fig. 5A, B; PERMANOVA; Bray–Curtis, *p* = 0.001; U.Unifrac, *p* = 0.001).

To identify shared water-gill bacteria at each site, the core microbiome of fish from each site was determined (85% ASV prevalence, n = 8 fish) and compared to the surrounding water ASVs from the same site. Only ASVs with an abundance > 0.1% were considered. Within hatcheries, a high percentage of the core gill microbiome was shared with the surrounding water (63.7–88.1%, Table 4). In general, there wasn’t a difference between the attached and the pelagic water fractions, with the exception of hatchery 6 (RAS) where only 16.4% of ASVs in the core gill were shared with the surrounding pelagic water fraction. All water and gill core ASVs from Hatchery 4 (Loch) and Hatchery 5 (FT) shared ASVs; ASV_14 *Burkholderiales*, ASV_26 *Candidatus_Planktophila*, ASV_27 *Candidatus_Fonsibacter*, ASV_29 *Polynucleobacter* (also present in H6 cores), ASV_34 *Limnohabitans*, ASV_64 *Candidatus_Methylopumilus*, ASV_126 *uncultured actinobacterium* and ASV_135 *Sediminibacterium* (Supplementary Table 4-spreadsheet). In comparison, ASV_111 *Bosea* and ASV_193 *Rhodoferax* were found in all gill and water cores from Hatchery 6 (RAS) and the water-attached and gill cores of Hatchery 3 (RAS).

**Table 4:** Comparison of shared ASVs from Core microbiomes of Gills (85% min. prev) with surrounding water portioned as attached and pelagic water microbiomes

Site	No. of core gill microbiome ASVs (85% min. prev.)	Overlapping ASVs (Gills Vs. Water)	No. of water core microbiome ASVs (50% min. prev.)
Hatchery 3-S1	42	37 Attached	88.1%
		*N/A*	*N/A*
Hatchery 4	51	37 Attached	72.5%
		26 Pelagic	70.6%
Hatchery 5	33	24 Attached	72.7%
		24 Pelagic	72.7%
Hatchery 6	67	11 Attached	16.4%
		42 Pelagic	62.7%
Farm C	21	5 Attached	23.8%
		6 Pelagic	28.6%
Farm G	18	12 Attached	66.7%
		10 Pelagic	55.6%
Farm H	35	12 Attached	34.3%
		12 Pelagic	34.3%
Farm E	52	4 Attached	7.7%
		2 Pelagic	3.8%

In contrast once the fish were at sea, the number of shared gill-water ASVs decreased, ranging from as little as 3.8% shared gill-pelagic water to as much as 66.7% shared gill-attached water), and was more variable between sites (Table 4). However, it should be bore in mind that the number of days the fish were at sea prior to sampling varied among sites between 7 and 25 days (Table 1). All seawater gill and water cores included ASV_5 *Rhodobacteraceae* (Supplementary Table 4-spreadsheet)*,* while 8 of the 9 core microbiomes from Farms C, H and G included ASV_17 *Clade_Ia,* all cores from Farms C, H and E included ASV_40 *SAR86_clade* per site (Supplementary Table 4-spreadsheet).

## Discussion

### Hatchery type was a driver of the gill microbiome, with the gill microbiome of fish from the same hatchery type more similar to each other

Hatchery type was a clear driver of gill microbiome composition. Fish from freshwater flow-through and two different loch systems had more similar gill microbiomes than fish from the three different RAS systems, despite the hatcheries being located in different geographical areas and sampled over different seasons (winter and spring). Further, freshwater appears to be a consistent driver as fish from the same RAS hatchery (H3) sampled in winter and then the following spring were more similar to each other than fish from other hatcheries. This supports a previous observation from two RAS and one FT system [34], that showed RAS systems to be more similar, but furthers the observation that the gill microbiome from FT and loch systems are more similar irrespective of location and/or time sampled. The water recirculation and biofiltration system in RAS hatcheries excert a strong influence over the water and fish microbiome [13, 34]. Although other factors, such as tank biofilms within hatcheries, also contributes to the hatchery microbial signature [34]. Indeed, the selective pressure of hatchery type on gill microbiome was further evidenced by findings on core microbiome. While amongst all hatcheries two genera, *Flavobacterium* and an uncultured *Fibrobacteraceae* were present, when considering similar hatchery type, the number of shared core microbiome genera increased*.* For RAS hatcheries six genera were shared, while for loch and FT there were nine, and for the first time five shared ASVs, including a *Flavobacterium* sp. and a *Shingomonas sp*. Interestingly, when comparing the ASVs of water and gills, no shared *Shingomonas sp* were found. *Flavobacterium* were consistently found in the gill microbiome of all fish, in the top 25 taxa at all freshwater and seawater sites. These bacteria are known to be present in a wide range of environments including freshwater, fish and aquaculture environments [27]. While the genera includes known fish pathogens *Flavobacterium psychrophilum* and *Flavobacterium columnare*, the majority of Flavobacterium form part of the normal host microbiota, but may be opportunistic pathogens causing disease under specific circumstances [27]. Interestingly a *Shingomonas* phylotype from the skin mucus microbiome of farmed Atlantic Salmon, was experimentally shown to be likely to use mucus as a nutrient source [36]. Within hatcheries, a high percentage of the microbiome from the surrounding water was shared with the gill microbiome, indicating this is a strong driver of the gill microbiome composition. Shared fish-water microbiome is expected and previously reported [24, 30–32, 42, 46], but the increase in the number of core taxa, especially the 5 ASVS that were shared among all fish reared in three different FT and loch systems (one collected in winter and two the following spring) indicates that this is not simply a transient event, but results in a strong selective pressure on gill microbiome composition, and that the gill microbiome environment and the mucus layer support their persistence. Hatchery waters may therefore offer potential practical applications to positively influence the gill microbiome of fish within hatchery stages.

### Once transferred to sea, the gill microbiomes of fish became more similar, but a signature of the hatchery remains up to at least 25 days post transfer

Fish, and therefore the gill microbiomes undergo significant environmental changes in the move from freshwater hatchery to sea. These changes are likely more abrupt than what occurs in wild populations that are exposed to a gradual change in environmental conditions [2, 24, 33, 49]. In addition to the obvious shift in salinity [37], there can be significant changes in other environmental factors such as pH (e.g. pH 6.6/6.8 at hatchery to up to pH 8.1 in seawater). The change from freshwater to seawater was, as expected, accompanied by changes in the microbial community composition, but also changes in species richness and evenness. Previously species richness and evenness have been reported to decrease on transfer to sea, followed by recovery after 4 weeks [29]. We showed that this is not always the case with both increases, decreases and no statistical change in species richness and evenness on transfer to sea. However, the fish in this study were sampled between days 7 and 25 post transfer, indicating we may have missed immediate changes, but there was still no specific trend associated with time after transfer (Fig. 2 and Supplementary Table 3). Multiple studies have indicated days at sea influences salmon microbiome diversity [28, 30, 42, 52] as does salinity [37]. The earliest sample days post-transfer in our study (day 7 and day 12) showed either no change or an increase, while those sampled after a minimum of two weeks did in general (but not always) show an increase in species richness. Once at sea, irrespective of hatchery source, an overall dramatic change in microbial community composition that is to be expected and often reported occurred [17, 28, 30, 31, 49] and the gill microbiomes of all fish become more similar (R^2^ = 0.197, *p* = 0.001). This is turn resulted in a larger shared core microbiome at sea, with 10 shared genera among all sites and two ASVs (ASV_12 *Psychrobacter* and ASV_3 *Pseudoalteromonas).* However, interestingly, a signature from the initial hatchery was preserved (R^2^ 0.119, *p* = 0.001), with loch and FT hatcheries clustering despite being transferred from distinct sites. Fish originating from RAS hatchery 2 and 6 clustered, while fish from RAS hatchery 3 that were transferred to different farms varied most. Could early life interactions between fish and bacteria within hatcheries have long term implications on the gill microbiome (and fish health) as reflected by the continued hatchery signature at sea? A growing body of evidence indicates early fish-environmental microbiome interactions program the hosts innate immune system with lasting effects [20–22]. For example, oyster larvae exposed to natural seawater microbiomes induced a long-term positive effect on the innate immune system that in turn altered the associated microbiome and continued to influence it long after the initial exposure period [20].

Shared core microbiome at sea have been reported [29], but not among different locations. This indicates there are consistencies within the gill microbiome, and indeed the amount of variability described by the PCoA plots, is very low (~ 5% for dimension 1 and 2). Of note while a shared core microbiome have been reported before from a Scottish seawater salmon farm in winter 2019 [29], the ASVs identified were different to those found in this study, belonging to *Chlamydiaceae*. While this was a different site and time, there were also differences in the approaches used (sampling, DNA extraction, PCR etc.) making direct comparisons difficult, and highlights the need for unification of approaches to gill microbiome studies to further understanding. While there was an increase in the number of shared taxa among fish from different sites at sea, the number of gill microbiome taxa shared with the surrounding water decreased and was more variable than in freshwater. This may indicate that seawater bacteria are less well adapted to the mucus layer than freshwater, and that the seawater mucus community assembly is more deterministic than stochastic [54]. Despite this, a single taxa, ASV_5 Rhodobacteraceae, was core to both gill microbiomes and water from all seawater sites. *Rhodobacteraceae* are widely distributed in marine and aquaculture environments and include beneficial bacteria with potential pre/probiotic functions [47].

### *Method recommendations:* need for a unified approach

A challenge with host-bacteria microbiome studies is that several of the commonly used bacterial 16S rRNA primer sets also amplify host DNA. As a result significant proportions of the amplicon library is host DNA and not bacteria. Many fish microbiome papers report undertaking a decontamination step in their analysis to remove contamination from eukaryotic sources [5, 41, 48], yet they often do not report what proportion of salmon sequences were removed. The need for a gold standard methodology in 16S rRNA microbiome fish research [51] is further highlighted by the presence of host contamination. Likely this affects community analysis, especially for rare and low abundant taxa, as much of the sequencing reads are used on host amplicons. In this study, the V1-V2 region of the 16S rRNA gene was amplified using primers F27 and R338 to reduce host contamination. This region was selected as it had the lowest percentage similarity with salmon 18S rRNA (F27 65% similarity; F338 72% similarity, Supplementary Table 2) compared with those more commonly used primer sets targeting the V3-V4 [12, 17, 31, 35] or V4 regions [10, 25, 28, 34] in previous fish microbiome studies. Common primers in these sets include F341 (77% similarity), F515 (100% similarity) and R806 (85% similarity) (Supplementary Table 2), higher matches than the primers chosen in this study. The approach was highly effective, with no salmon 18S rRNA genes amplified. To improve fish host-microbiome results and encourage consistency in data generation of studies to facilitate more direct comparison among data sets and deepen our understanding, we recommend that this primer is widely adopted.

## Conclusion

Here we have shown that the gill microbiome from freshwater hatcheries and seawater farms at seven sites in Scotland, sampled over two different years and input seasons, have some remarkable trends and similarities. Hatchery type strongly selects for the gill microbiome, with the gills of fish from different loch and flow through systems highly similar to each other while RAS the gill microbiomes from different RAS systems are highly similar. Further there were more shared gill-water phylotypes by hatchery type, indicating the influence of the surrounding water microbiome. Once transferred to sea, the gill microbiomes of all fish became more similar irrespective of hatchery type, season of transfer or farm location. Despite becoming more similar at sea, the number of shared ASVs between the gill microbiome and surrounding water reduced, and some clustering by the hatchery of origin was evident. It remains to be seen if this signature from hatchery persists over time at sea, but if it does, hatchery waters may offer opportunities to positively influence the gill microbiome of fish with lasting benefits during their life at sea.

## Supplementary Information

Additional file 1.

Additional file 2.

Additional file 3.

## Data Availability

Raw sequence files supporting the results of this article are available in the European Nucleotide Archive under the project accession number PRJEB65363.

## References

[1] Amann RI, Binder BJ, Olson RJ, Chisholm SW, Devereux R, Stahl DA. Combination of 16S rRNA-targeted oligonucleotide probes with flow cytometry for analyzing mixed microbial populations. Appl Environ Microbiol. 1990;56:1919–25. 10.1128/aem.56.6.1919-1925.1990. 2200342 10.1128/aem.56.6.1919-1925.1990 PMC184531

[2] Amill F, Gauthier J, Rautio M, Derome N. Characterization of gill bacterial microbiota in wild Arctic char ( *Salvelinus alpinus* ) across lakes, rivers, and bays in the Canadian Arctic ecosystems. Microbiol Spectr. 2024;12:e02943-e3023. 10.1128/spectrum.02943-23. 38329329 10.1128/spectrum.02943-23 PMC10923216

[3] Atencio LA, Dal Grande F, Young GO, Gavilán R, Guzmán HM, Schmitt I, Mejía LC, Gutiérrez M. Antimicrobial-producing *Pseudoalteromonas* from the marine environment of Panama shows a high phylogenetic diversity and clonal structure. J Basic Microbiol. 2018;58:747–69. 10.1002/jobm.201800087. 29938809 10.1002/jobm.201800087

[4] Bergheim A, Drengstig A, Ulgenes Y, Fivelstad S. Production of Atlantic salmon smolts in Europe—Current characteristics and future trends. Aquacult Eng. 2009;41:46–52. 10.1016/j.aquaeng.2009.04.004.

[5] Birlanga VB, McCormack G, Ijaz U.Z, McCarthy E, Smith C, Collins G. Dynamic gill and mucous microbiomes track an amoebic gill disease episode in farmed Atlantic salmon (preprint). In review 2020 10.21203/rs.3.rs-29747/v1 10.1038/s41598-022-17008-2 PMC9537138 36202859

[6] Boerlage AS, Ashby A, Herrero A, Reeves A, Gunn GJ, Rodger HD. Epidemiology of marine gill diseases in Atlantic salmon (*Salmo salar*) aquaculture: a review. Rev Aquacult. 2020;12:2140–59. 10.1111/raq.12426.

[7] Bowman JP, Nowak B. Salmonid gill bacteria and their relationship to amoebic gill disease. J Fish Diseases. 2004;27:483–92. 10.1111/j.1365-2761.2004.00569.x. 15291790 10.1111/j.1365-2761.2004.00569.x

[8] Brown R, Moore L, Mani A, Patel S, Salinas I. Effects of ploidy and salmonid alphavirus infection on the skin and gill microbiome of Atlantic salmon (*Salmo salar*). PLoS ONE. 2021;16: e0243684. 10.1371/journal.pone.0243684. 33606747 10.1371/journal.pone.0243684 PMC7894865

[9] Brown RM, Wiens GD, Salinas I. Analysis of the gut and gill microbiome of resistant and susceptible lines of rainbow trout (*Oncorhynchus mykiss*). Fish Shellfish Immunol. 2019;86:497–506. 10.1016/j.fsi.2018.11.079. 30513381 10.1016/j.fsi.2018.11.079 PMC8040288

[10] Bugten AV, Attramadal KJK, Fossmark RO, Rosten TW, Vadstein O, Bakke I. Changes in rearing water microbiomes in RAS induced by membrane filtration alters the hindgut microbiomes of Atlantic salmon (*Salmo salar*) parr. Aquaculture. 2022;548: 737661. 10.1016/j.aquaculture.2021.737661.

[11] Callahan BJ, McMurdie PJ, Rosen MJ, Han AW, Johnson AJA, Holmes SP. DADA2: High-resolution sample inference from Illumina amplicon data. Nat Methods. 2016;13:581–3. 10.1038/nmeth.3869. 27214047 10.1038/nmeth.3869 PMC4927377

[12] Clinton M, Wyness AJ, Martin SAM, Brierley AS, Ferrier DEK. Sampling the fish gill microbiome: a comparison of tissue biopsies and swabs. BMC Microbiol. 2021;21:313. 10.1186/s12866-021-02374-0. 34758745 10.1186/s12866-021-02374-0 PMC8579561

[13] Dahle SW, Gaarden SI, Buhaug JF, Netzer R, Attramadal KJK, Busche T, Aas M, Ribicic D, Bakke I. Long-term microbial community structures and dynamics in a commercial RAS during seven production batches of Atlantic salmon fry (*Salmo salar*). Aquaculture. 2023;565: 739155. 10.1016/j.aquaculture.2022.739155.

[14] Dalsgaard J, Lund I, Thorarinsdottir R, Drengstig A, Arvonen K, Pedersen PB. Farming different species in RAS in Nordic countries: current status and future perspectives. Aquacult Eng. 2013;53:2–13. 10.1016/j.aquaeng.2012.11.008.

[15] Davis NM, Proctor DM, Holmes SP, Relman DA, Callahan BJ. Simple statistical identification and removal of contaminant sequences in marker-gene and metagenomics data. Microbiome. 2018;6:226. 10.1186/s40168-018-0605-2. 30558668 10.1186/s40168-018-0605-2 PMC6298009

[16] de Bruijn I, Liu Y, Wiegertjes GF, Raaijmakers JM. Exploring fish microbial communities to mitigate emerging diseases in aquaculture. FEMS Microbiology Ecology. 2018. 10.1093/femsec/fix161. 29206925 10.1093/femsec/fix161

[17] Dehler CE, Secombes CJ, Martin SAM. Seawater transfer alters the intestinal microbiota profiles of Atlantic salmon (*Salmo salar* L.). Sci Rep. 2017;7:13877. 10.1038/s41598-017-13249-8. 29066818 10.1038/s41598-017-13249-8 PMC5654775

[18] Downes JK, Yatabe T, Marcos-Lopez M, Rodger HD, MacCarthy E, O’Connor I, Collins E, Ruane NM. Investigation of co-infections with pathogens associated with gill disease in Atlantic salmon during an amoebic gill disease outbreak. J Fish Dis. 2018;41:1217–27. 10.1111/jfd.12814. 10.1111/jfd.12814 29806080

[19] Egan S, Gardiner M. Microbial Dysbiosis: rethinking disease in marine ecosystems. Front Microbiol. 2016. 10.3389/fmicb.2016.00991. 27446031 10.3389/fmicb.2016.00991 PMC4914501

[20] Fallet M, Montagnani C, Petton B, Dantan L, De Lorgeril J, Comarmond S, Chaparro C, Toulza E, Boitard S, Escoubas J-M, Vergnes A, Le Grand J, Bulla I, Gueguen Y, Vidal-Dupiol J, Grunau C, Mitta G, Cosseau C. Early life microbial exposures shape the Crassostrea gigas immune system for lifelong and intergenerational disease protection. Microbiome. 2022;10:85. 10.1186/s40168-022-01280-5. 35659369 10.1186/s40168-022-01280-5 PMC9167547

[21] Galindo-Villegas J, García-Moreno D, De Oliveira S, Meseguer J, Mulero V. Regulation of immunity and disease resistance by commensal microbes and chromatin modifications during zebrafish development. Proc Natl Acad Sci USA. 2012. 10.1073/pnas.1209920109. 22949679 10.1073/pnas.1209920109 PMC3465450

[22] Gomez D, Sunyer JO, Salinas I. The mucosal immune system of fish: the evolution of tolerating commensals while fighting pathogens. Fish Shellfish Immunol. 2013;35:1729–39. 10.1016/j.fsi.2013.09.032. 24099804 10.1016/j.fsi.2013.09.032 PMC3963484

[23] Herrero A, Thompson KD, Ashby A, Rodger HD, Dagleish MP. Complex gill disease: an emerging syndrome in farmed atlantic salmon (*Salmo salar* L.). J Comp Pathol. 2018;163:23–8. 10.1016/j.jcpa.2018.07.004. 30213370 10.1016/j.jcpa.2018.07.004

[24] Itay P, Shemesh E, Ofek-Lalzar M, Davidovich N, Kroin Y, Zrihan S, Stern N, Diamant A, Wosnick N, Meron D, Tchernov D, Morick D. An insight into gill microbiome of Eastern Mediterranean wild fish by applying next generation sequencing. Front Mar Sci. 2022;9:1008103. 10.3389/fmars.2022.1008103.

[25] Keating C, Bolton-Warberg M, Hinchcliffe J, Davies R, Whelan S, Wan AHL, Fitzgerald RD, Davies SJ, Ijaz UZ, Smith CJ. Temporal changes in the gut microbiota in farmed Atlantic cod (*Gadus morhua*) outweigh the response to diet supplementation with macroalgae. Anim Microbiome. 2021. 10.1186/s42523-020-00065-1. 33500003 10.1186/s42523-020-00065-1 PMC7934267

[26] Leo Lahti et al. (Bioconductor, 2017). Tools for microbiome analysis in R. Microbiome package version. URL: (http://microbiome.github.io/microbiome)

[27] Lee B-H, Nicolas P, Saticioglu IB, Fradet B, Bernardet J-F, Rigaudeau D, Rochat T, Duchaud E. Investigation of the genus *Flavobacterium* as a reservoir for fish-pathogenic bacterial species: the case of Flavobacterium collinsii. Appl Environ Microbiol. 2023;89:e02162-e2222. 10.1128/aem.02162-22. 36975784 10.1128/aem.02162-22 PMC10132118

[28] Lokesh J, Kiron V. Transition from freshwater to seawater reshapes the skin-associated microbiota of Atlantic salmon. Sci Rep. 2016;6:19707. 10.1038/srep19707. 26806545 10.1038/srep19707 PMC4726331

[29] Lorgen-Ritchie M, Chalmers L, Clarkson M, Taylor JF, Migaud H, Martin SAM. Time is a stronger predictor of microbiome community composition than tissue in external mucosal surfaces of Atlantic salmon (*Salmo salar*) reared in a semi-natural freshwater environment. Aquaculture. 2023;566: 739211. 10.1016/j.aquaculture.2022.739211.

[30] Lorgen-Ritchie M, Clarkson M, Chalmers L, Taylor JF, Migaud H, Martin SAM. Temporal changes in skin and gill microbiomes of Atlantic salmon in a recirculating aquaculture system—Why do they matter? Aquaculture. 2022;558: 738352. 10.1016/j.aquaculture.2022.738352.

[31] Lorgen-Ritchie M, Clarkson M, Chalmers L, Taylor JF, Migaud H, Martin SAM. A temporally dynamic gut microbiome in atlantic salmon during freshwater recirculating aquaculture system (RAS) production and post-seawater transfer. Front Mar Sci. 2021;8: 711797. 10.3389/fmars.2021.711797.

[32] Mes W, Lücker S, Jetten MSM, Siepel H, Gorissen M, Van Kessel MAHJ. Comparison of the gill and gut microbiomes of common carp (*Cyprinus carpio*) and zebrafish (*Danio rerio*) and their RAS environment. Sci Total Environ. 2023;896: 165212. 10.1016/j.scitotenv.2023.165212. 37391154 10.1016/j.scitotenv.2023.165212

[33] Minich JJ, Petrus S, Michael JD, Michael TP, Knight R, Allen EE. Temporal, environmental, and biological drivers of the mucosal microbiome in a wild marine fish *Scomber japonicus*. MSphere. 2020. 10.1128/mSphere.00401-20. 32434844 10.1128/mSphere.00401-20 PMC7380571

[34] Minich JJ, Poore GD, Jantawongsri K, Johnston C, Bowie K, Bowman J, Knight R, Nowak B, Allen EE. Microbial ecology of Atlantic salmon (*Salmo salar*) Hatcheries: impacts of the built environment on fish Mucosal Microbiota. Appl Environ Microbiology. 2020;86:19. 10.1128/AEM.00411-20 PMC7267192 32303543

[35] Minniti G, Hagen LH, Porcellato D, Jørgensen SM, Pope PB, Vaaje-Kolstad G. The skin-mucus microbial community of farmed Atlantic salmon (*Salmo salar*). Front Microbiol. 2017;8:2043. 10.3389/fmicb.2017.02043. 29104567 10.3389/fmicb.2017.02043 PMC5655796

[36] Minniti G, Rød Sandve S, Padra JT, Heldal Hagen L, Lindén S, Pope PB, Arntzen ØM, Vaaje-Kolstad G. The farmed Atlantic salmon (*Salmo salar*) skin-mucus proteome and its nutrient potential for the resident bacterial community. Genes. 2019;10:515. 10.3390/genes10070515. 31284681 10.3390/genes10070515 PMC6678340

[37] Morales-Rivera MF, Valenzuela-Miranda D, Nuñez-Acuña G, Benavente BP, Gallardo-Escárate C, Valenzuela-Muñoz V. Atlantic salmon (*Salmo salar*) transfer to seawater by gradual salinity changes exhibited an increase in the intestinal microbial abundance and richness. Microorganisms. 2022;11:76. 10.3390/microorganisms11010076. 36677368 10.3390/microorganisms11010076 PMC9865641

[38] Munro LA. Scottish Fish Farm Production Survey 2022. 2023

[39] Offret C, Desriac F, Le Chevalier P, Mounier J, Jégou C, Fleury Y. Spotlight on antimicrobial metabolites from the marine bacteria pseudoalteromonas: chemodiversity and ecological significance. Mar Drugs. 2016;14:129. 10.3390/md14070129. 27399731 10.3390/md14070129 PMC4962019

[40] Jari Oksanen F, G Blanchet, M Friendly, R Kindt, P Legendre, D McGlinn, PR. Minchin, R. B. O'Hara, GL Simpson, P Solymos, M Henry, H Stevens, E Szoecs, H Wagner. vegan: community ecology package. R package version 2.5-7 2020 https://CRAN.R-project.org/package=vegan

[41] Palladino G, Rampelli S, Scicchitano D, Musella M, Quero GM, Prada F, Mancuso A, Seyfarth AM, Turroni S, Candela M, Biagi E. Impact of marine aquaculture on the microbiome associated with nearby Holobionts: the case of patella Caerulea living in proximity of sea bream aquaculture cages. Microorganisms. 2021;9:455. 10.3390/microorganisms9020455. 33671759 10.3390/microorganisms9020455 PMC7927081

[42] Quezada-Rodriguez PR, Downes J, Egan F, Taylor RS, White S, Brenan A, Rigby ML, Nowak BF, Wynne JW, Ruane NM. Assessment of gill microbiome of two strains of Atlantic salmon reared in flowthrough and recirculation hatcheries and following seawater transfer. Aquaculture. 2024;580: 740322. 10.1016/j.aquaculture.2023.740322.

[43] Quezada-Rodriguez PR, Taylor RS, Samsing F, Rigby M, Wood AT, Nowak BF, Wynne JW. Effect of a prophylactic treatment with chloramine-T on gill histology and microbiome of Atlantic salmon (*Salmo salar*) under commercial conditions. Aquaculture. 2022;546: 737319. 10.1016/j.aquaculture.2021.737319.

[44] Rudi K, Angell IL, Pope PB, Vik JO, Sandve SR, Snipen L-G. Stable core gut microbiota across the freshwater-to-saltwater transition for farmed Atlantic salmon. Appl Environ Microbiol. 2018. 10.1128/AEM.01974-17. 29101198 10.1128/AEM.01974-17 PMC5752857

[45] Schmieder R, Edwards R. Fast identification and removal of sequence contamination from genomic and metagenomic datasets. PLoS ONE. 2011;6: e17288. 10.1371/journal.pone.0017288. 21408061 10.1371/journal.pone.0017288 PMC3052304

[46] Smith CJ, Danilowicz BS, Meijer WG. Characterization of the bacterial community associated with the surface and mucus layer of whiting (*Merlangius merlangus*): bacterial community associated with whiting mucus layer. FEMS Microbiol Ecol. 2007;62:90–7. 10.1111/j.1574-6941.2007.00369.x. 17692096 10.1111/j.1574-6941.2007.00369.x

[47] Sonnenschein EC, Jimenez G, Castex M, Gram L. The *Roseobacter* -group bacterium *Phaeobacter* as a safe probiotic solution for aquaculture. Appl Environ Microbiol. 2021;87:e02581-e2620. 10.1128/AEM.02581-20. 33310713 10.1128/AEM.02581-20 PMC8090895

[48] Steiner K, Heasman K, Laroche O, Pochon X, Preece M, Bowman JP, Walker SP, Symonds JE. The microbiome of Chinook salmon (*Oncorhynchus tshawytscha*) in a recirculation aquaculture system. Aquaculture. 2021;534: 736227. 10.1016/j.aquaculture.2020.736227.

[49] Uren Webster TM, Rodriguez-Barreto D, Castaldo G, Gough P, Consuegra S, Garcia de Leaniz C. Environmental plasticity and colonisation history in the Atlantic salmon microbiome: a translocation experiment. Mol Ecol. 2020;29:886–98. 10.1111/mec.15369. 32011775 10.1111/mec.15369 PMC7078932

[50] van Kessel MAHJ, Mesman RJ, Arshad A, Metz JR, Spanings FAT, van Dalen SCM, van Niftrik L, Flik G, Wendelaar Bonga SE, Jetten MSM, Klaren PHM, Op den Camp HJM. Branchial nitrogen cycle symbionts can remove ammonia in fish gills. Environ Microbiol Rep. 2016;8:590–4. 10.1111/1758-2229.12407. 27040730 10.1111/1758-2229.12407

[51] Vatsos IN. Standardizing the microbiota of fish used in research. Lab Anim. 2017;51:353–64. 10.1177/0023677216678825. 27932684 10.1177/0023677216678825

[52] Wang J, Jaramillo-Torres A, Li Y, Kortner TM, Gajardo K, Brevik ØJ, Jakobsen JV, Krogdahl Å. Microbiota in intestinal digesta of Atlantic salmon (*Salmo salar*), observed from late freshwater stage until one year in seawater, and effects of functional ingredients: a case study from a commercial sized research site in the Arctic region. anim microbiome. 2021. 10.1186/s42523-021-00075-7. 33509296 10.1186/s42523-021-00075-7 PMC7841887

[53] Yu Y, Ding L, Huang Z, Xu H, Xu Z. Commensal bacteria-immunity crosstalk shapes mucosal homeostasis in teleost fish. Rev Aquacult. 2021;13:2322–43. 10.1111/raq.12570.

[54] Zhou J, Ning D. Stochastic community assembly: Does it matter in microbial ecology? Microbiol Mol Biol Rev. 2017;81:e00002-17. 10.1128/MMBR.00002-17. 29021219 10.1128/MMBR.00002-17 PMC5706748

