# Supplementary material for: Hatchery type influences the gill microbiome of Atlantic farmed salmon (Salmo salar) after transfer to sea
Source: Anim Microbiome. 2024 Nov 8;6:65. doi: 10.1186/s42523-024-00347-y (PMC11549768; doi:10.1186/s42523-024-00347-y)

**Supplementary Files**

**Hatchery type influences the gill microbiome of Atlantic farmed salmon (*Salmo salar*) after transfer to sea**

**Authors**; Kelly J. Stewart^1^, Annette S. Boerlage^2^, William Barr^1^, Umer Z. Ijaz^1^, Cindy J. Smith^1*^

**Affiliations**; 1. Infrastructure and Environment, James Watt School of Engineering, University of Glasgow, Glasgow, Scotland, UK.

2. Centre for Epidemiology and Planetary Health (CEPH), SRUC School of Veterinary Medicine, Scotland’s Rural College (SRUC), Inverness, UK

**Supplementary Table 1. Primer sequences.** Primers adapted from Fadrosh et al. (2014), using primers F27 (AGAGTTTGATCMTGGCTCAG) (Yuan et al., 2012) and R338 (5’ -GCTGCCTCCCGTAGGAGT) (Amann et al., 1990)

| Primer name | Sequence linker 1 or 2 | Index | Heterogeneity  Spacer | Primer  (5’) |
| --- | --- | --- | --- | --- |
| F27-01 | **1** | CCTAAACTACGG | *n/a* | AGAGTTTGATCMTGGCTCAG |
| F27-02 | **1** | TGCAGATCCAAC | *n/a* | AGAGTTTGATCMTGGCTCAG |
| F27-03 | **1** | CCATCACATAGG | *n/a* | AGAGTTTGATCMTGGCTCAG |
| F27-04 | **1** | GTGGTATGGGAG | T | AGAGTTTGATCMTGGCTCAG |
| F27-05 | **1** | ACTTTAAGGGTG | T | AGAGTTTGATCMTGGCTCAG |
| F27-06 | **1** | GAGCAACATCCT | T | AGAGTTTGATCMTGGCTCAG |
| F27-07 | **1** | TGTTGCGTTTCT | GT | AGAGTTTGATCMTGGCTCAG |
| F27-08 | **1** | ATGTCCGACCAA | GT | AGAGTTTGATCMTGGCTCAG |
| F27-09 | **1** | AGGTACGCAATT | GT | AGAGTTTGATCMTGGCTCAG |
| F27-10 | **1** | ACAGCCACCCAT | CGA | AGAGTTTGATCMTGGCTCAG |
| F27-11 | **1** | TGTCTCGCAAGC | CGA | AGAGTTTGATCMTGGCTCAG |
| F27-12 | **1** | GAGGAGTAAAGC | CGA | AGAGTTTGATCMTGGCTCAG |
| F27-13 | **1** | GTTACGTGGTTG | ATGA | AGAGTTTGATCMTGGCTCAG |
| F27-14 | **1** | TACCGCCTCGGA | ATGA | AGAGTTTGATCMTGGCTCAG |
| F27-15 | **1** | CGTAAGATGCCT | ATGA | AGAGTTTGATCMTGGCTCAG |
| R338-01 | **2** | CCTAAACTACGG | *n/a* | GCTGCCTCCCGTAGGAGT |
| R338-02 | **2** | TGCAGATCCAAC | *n/a* | GCTGCCTCCCGTAGGAGT |
| R338-03 | **2** | CCATCACATAGG | *n/a* | GCTGCCTCCCGTAGGAGT |
| R338-04 | **2** | GTGGTATGGGAG | A | GCTGCCTCCCGTAGGAGT |
| R338-05 | **2** | ACTTTAAGGGTG | A | GCTGCCTCCCGTAGGAGT |
| R338-06 | **2** | GAGCAACATCCT | A | GCTGCCTCCCGTAGGAGT |
| R338-07 | **2** | TGTTGCGTTTCT | TC | GCTGCCTCCCGTAGGAGT |
| R338-08 | **2** | ATGTCCGACCAA | TC | GCTGCCTCCCGTAGGAGT |
| R338-09 | **2** | AGGTACGCAATT | TC | GCTGCCTCCCGTAGGAGT |
| R338-10 | **2** | ACAGCCACCCAT | CTA | GCTGCCTCCCGTAGGAGT |
| R338-11 | **2** | TGTCTCGCAAGC | CTA | GCTGCCTCCCGTAGGAGT |
| R338-12 | **2** | GAGGAGTAAAGC | CTA | GCTGCCTCCCGTAGGAGT |

**Linker sequence 1 (Forward primers) =** 5’CAAGCAGAAGACGGCATACGAGATGTGACTGGAGTTCAGACGTGTGCTCTTCCGATCT

**Linker sequence 2 (Reverse primers) =** 5’AATGATACGGCGACCACCGAGATCTACACTCTTTCCCTACACGACGCTCTTCCGATCT

**Supplementary Table 2. Universal 16S rRNA gene primers sequences alignment to 18S rRNA gene of Atlantic salmon (*Salmo salar*).** Alignment was performed using ClustalX2 software on mac (Larkin et al., 2007), aligning each primer sequence to the reference 18S rRNA gene sequence (European Nucleotide Archive 2019, Accession; FJ710886).

| Primer | % similarity to 18S salmon | Reference |
| --- | --- | --- |
| **F27** | **65%** | (Lane et al., 1991, Yuan et al., 2012) |
| F63 | 70% | (Beckers et al., 2016; Thijs et al., 2017) |
| **R338** | **72%** | (Amann et al., 1990; Salter et al., 2014) |
| F341 | 77% | (Dehler et al., 2017; Minniti et al., 2017; Takahashi et al., 2014) |
| F515 | 100% | Earth Microbiome (Parada et al., 2016) |
| R518 | 100% | (Thijs et al., 2017) |
| R534 | 100% | (Walker et al., 2015) |
| R785 | 83% | (Llewellyn et al., 2017) |
| R806 | 85% | Earth Microbiome (Parada et al., 2016) |
| R926 | 100% | Earth Microbiome (Parada et al., 2016) |
| F1369 | 60% | (Suzuki et al., 2000) |
| Probe1389 | 95% | (Suzuki et al., 2000) |
| R1492 | 47% | (Suzuki et al., 2000) |


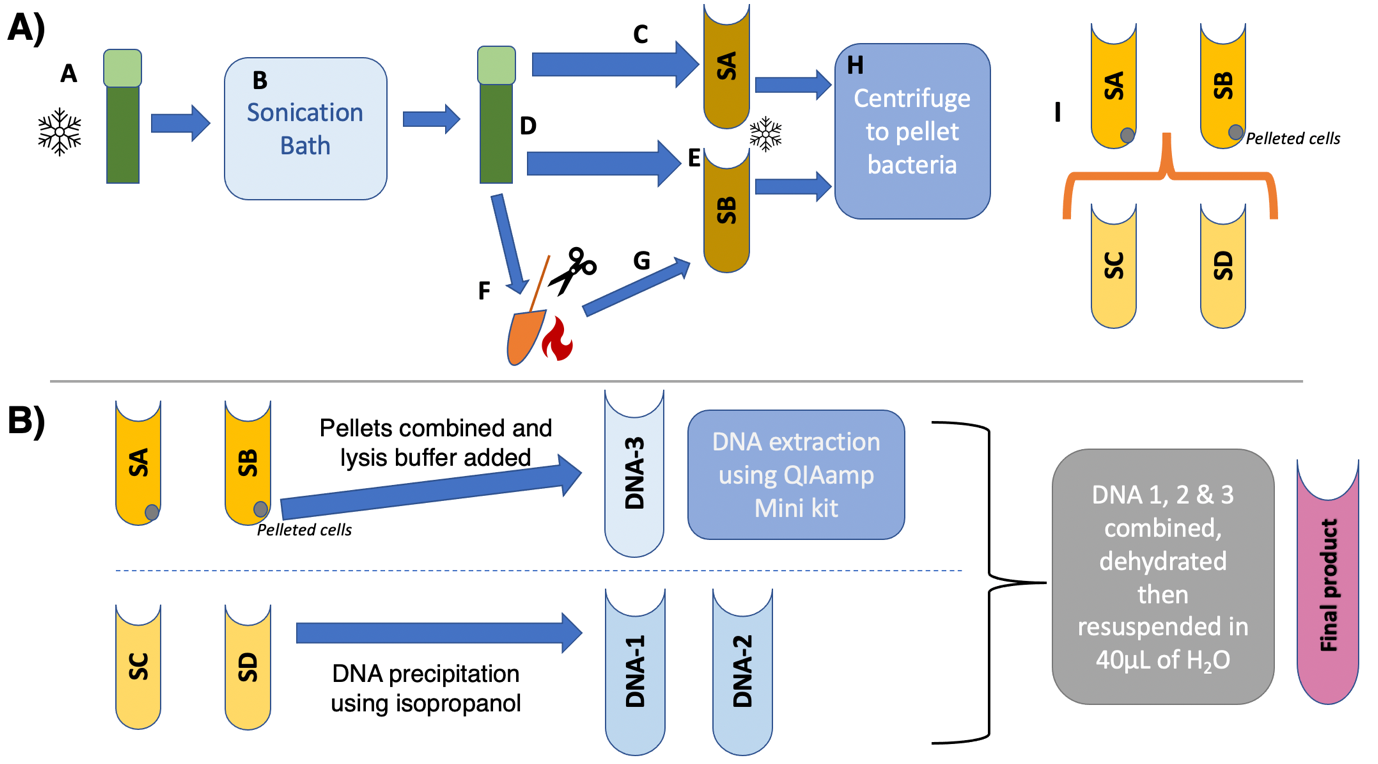
**Supplementary Figure 1. Preparation of mucosal gill swabs for DNA extraction**. **A)** eNAT swabs are defrosted on ice (A), then transferred to a sonication bath for 2 minutes are 40kHz (B). The swabs were centrifuged for a minute at 3000rpm, and the supernatant transferred to a sterile 1.5ml Eppendorf tube (SA, step C). Following this, 800μL of PBS was added to the empty tube (D), pressing the head of the swab against the sides of the tube to detach any remaining cells. The tube was then vortexed at 1200rpm for 10seconds, quickly centrifuged then the supernatant was transferred to a sterile 1.5ml Eppendorf tube (SB, step E). The swab heads were then cut from their plastic stems (F) and transferred to sterile 1.5mL tubes containing cut pipette tips and spun for 1 minute at max speed to collect the remaining supernatant, adding this to the applicable existing supernatant tube (SB, step G), repeating till the swab is dry. The two collected tubes (SA and SB) were then centrifuged at 12,000g for 20 minutes at 4oC to create a pellet of the bacterial cells (H). The supernatant of these two tubes were transferred to sterile 2.0mL tubes (SC & SD, step I), leaving the pelleted cells behind in their respective tubes (SA & SB, step I). **B)** Pellets from tubes 1 & 2 are combined and resuspended in the lysis buffer, then DNA extraction is carried out using the QIAamp Mini kit (DNA-1). DNA in S3 & S4 is precipitated using an isopropanol method (Green & Sambrook 2016) creating DNA-2 and DNA-3 elution’s. The three extract tubes are then combined, dehydrated using the Effendorf Concentrator plus (UK) by running the V-AQ mode at 45oC and resuspended in 40μL of DNase free water.

**Supplementary Figure 2.** **Most abundant Phylum of farmed Atlantic salmon.** Each bar represents an individual salmon gill microbiome, plotting fish 5-8 from unit A, then fish 5-8 from unit B in series. Samples grouped by site, with each column representing an individual fish.


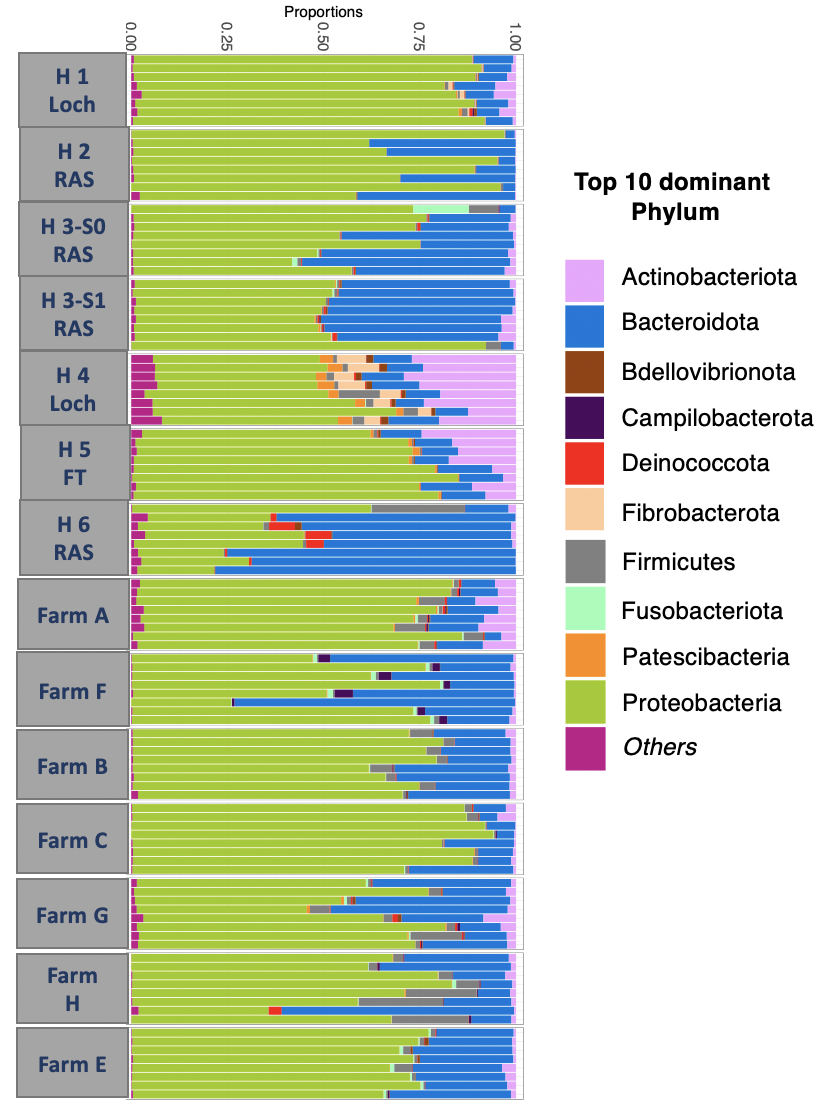


**Supplementary Figure 3.** **Core microbiome (85% min. prevalence) heat maps of freshwater and seawater mucosal gill microbiomes at genus and ASV level for relative abundances.** **A)** Core microbiome of all salmon gills at genus level **B)** Core microbiome of freshwater only salmon gills at genus level **C)** Core microbiome of Seawater salmon gills at genus level **D)** Core microbiome of seawater salmon gills at ASV level.


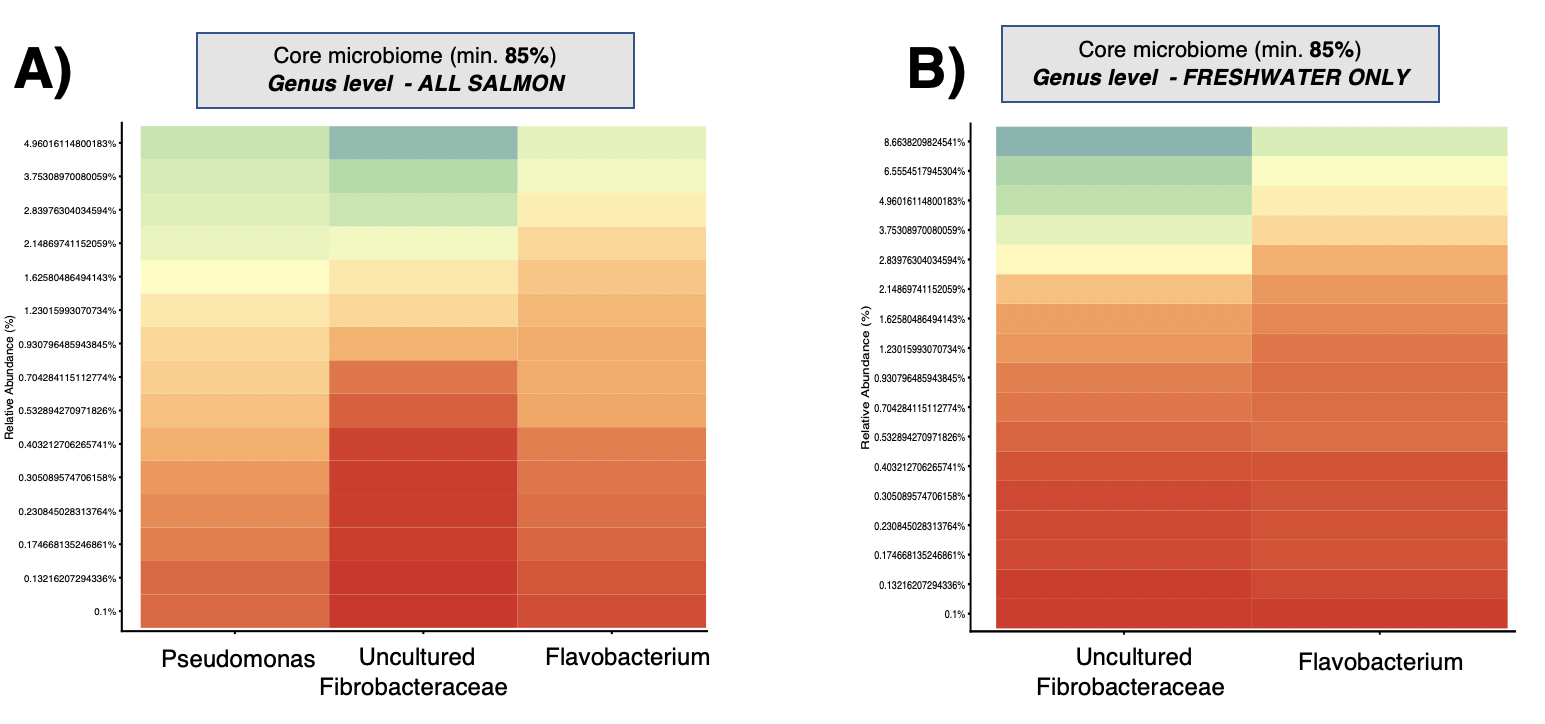

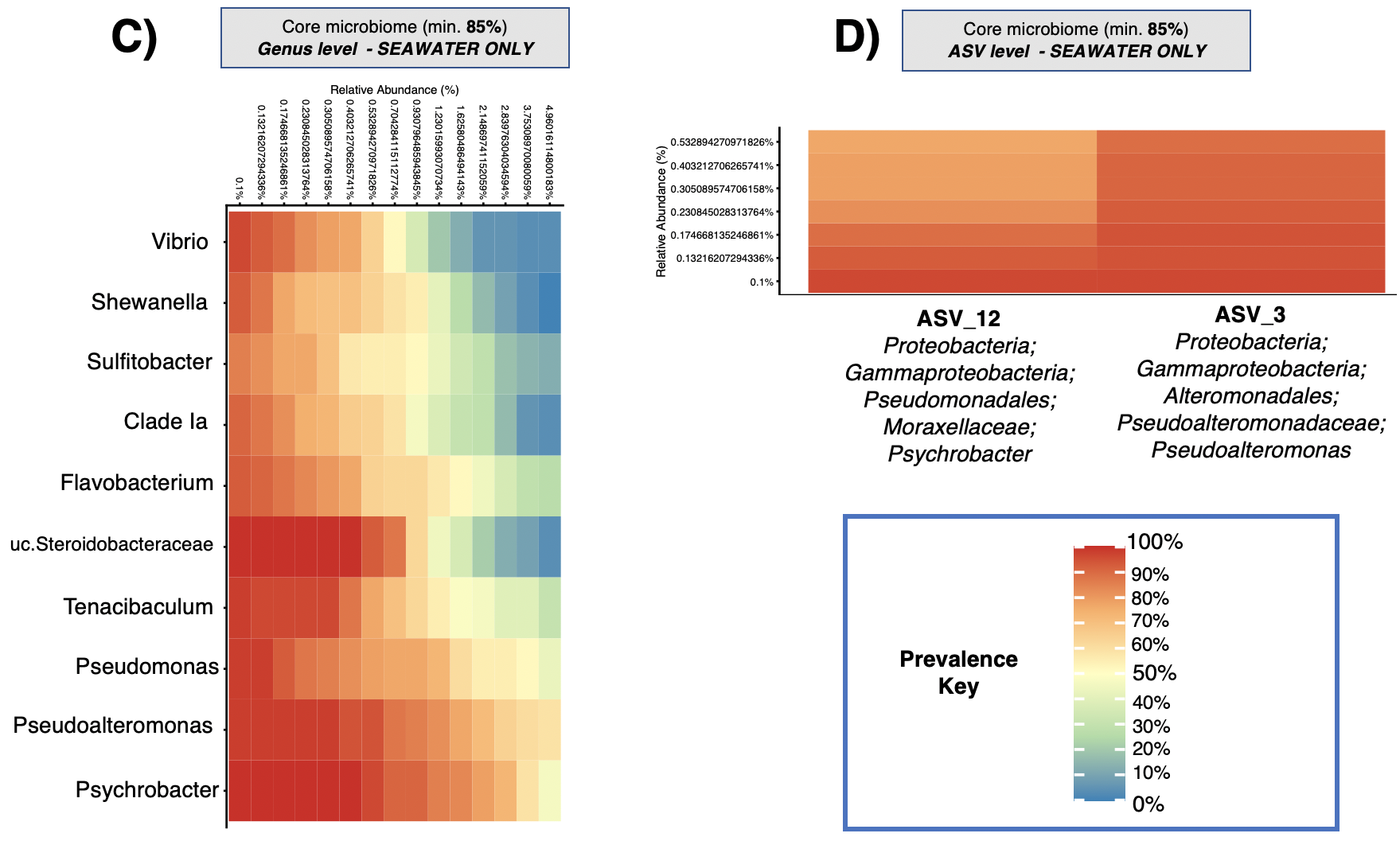


**Supplementary Figure 4.** **Core microbiome (85% min. prevalence) heat maps of freshwater mucosal gill microbiomes at genus and ASV level for relative abundances, grouped by hatchery types.** **A)** Core microbiome of all RAS hatchery gills at genus level **B)** Core microbiome of Loch and FT hatchery gills at genus level **C)** Core microbiome of Loch and FT hatchery gills at ASV level


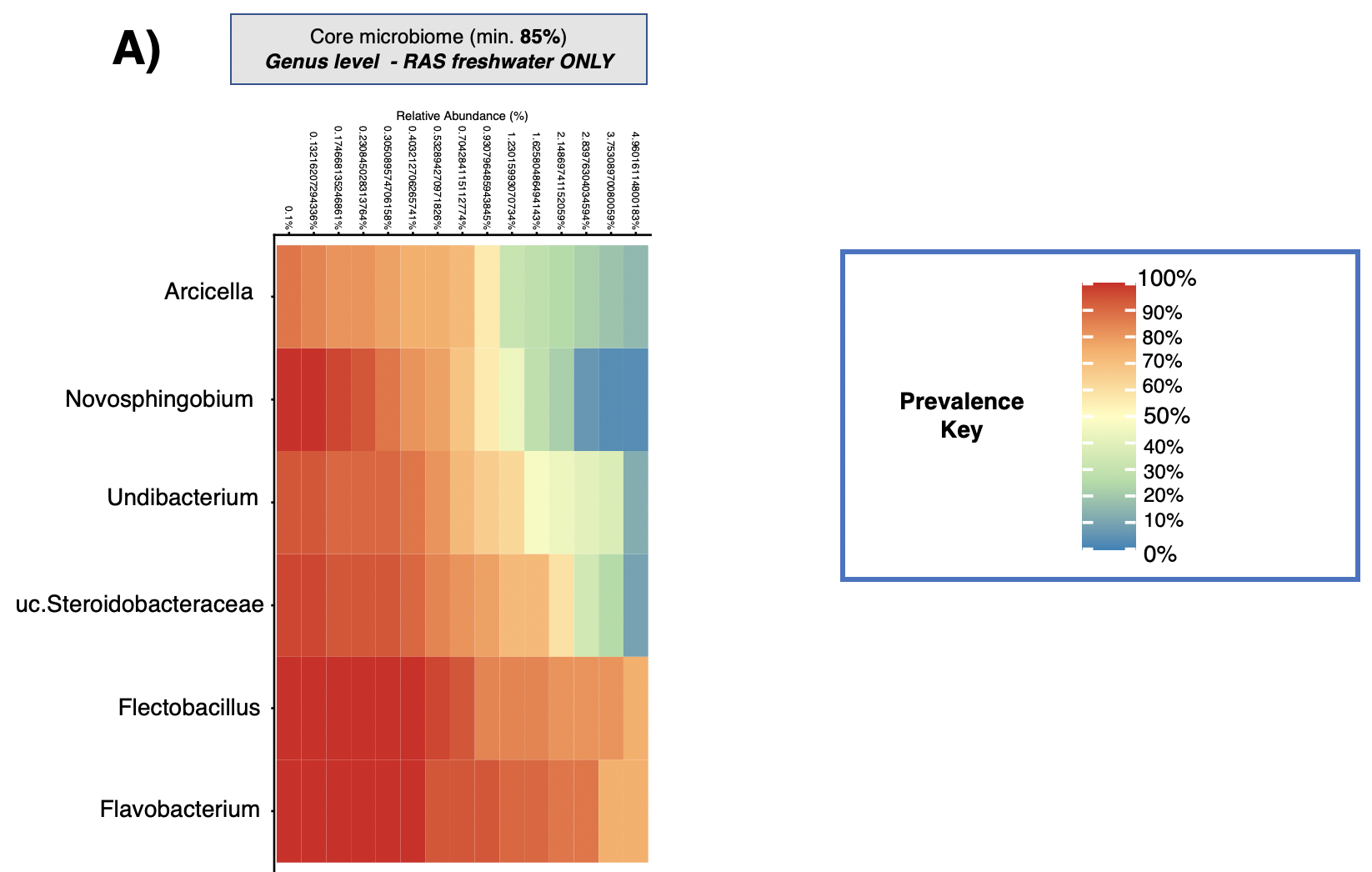


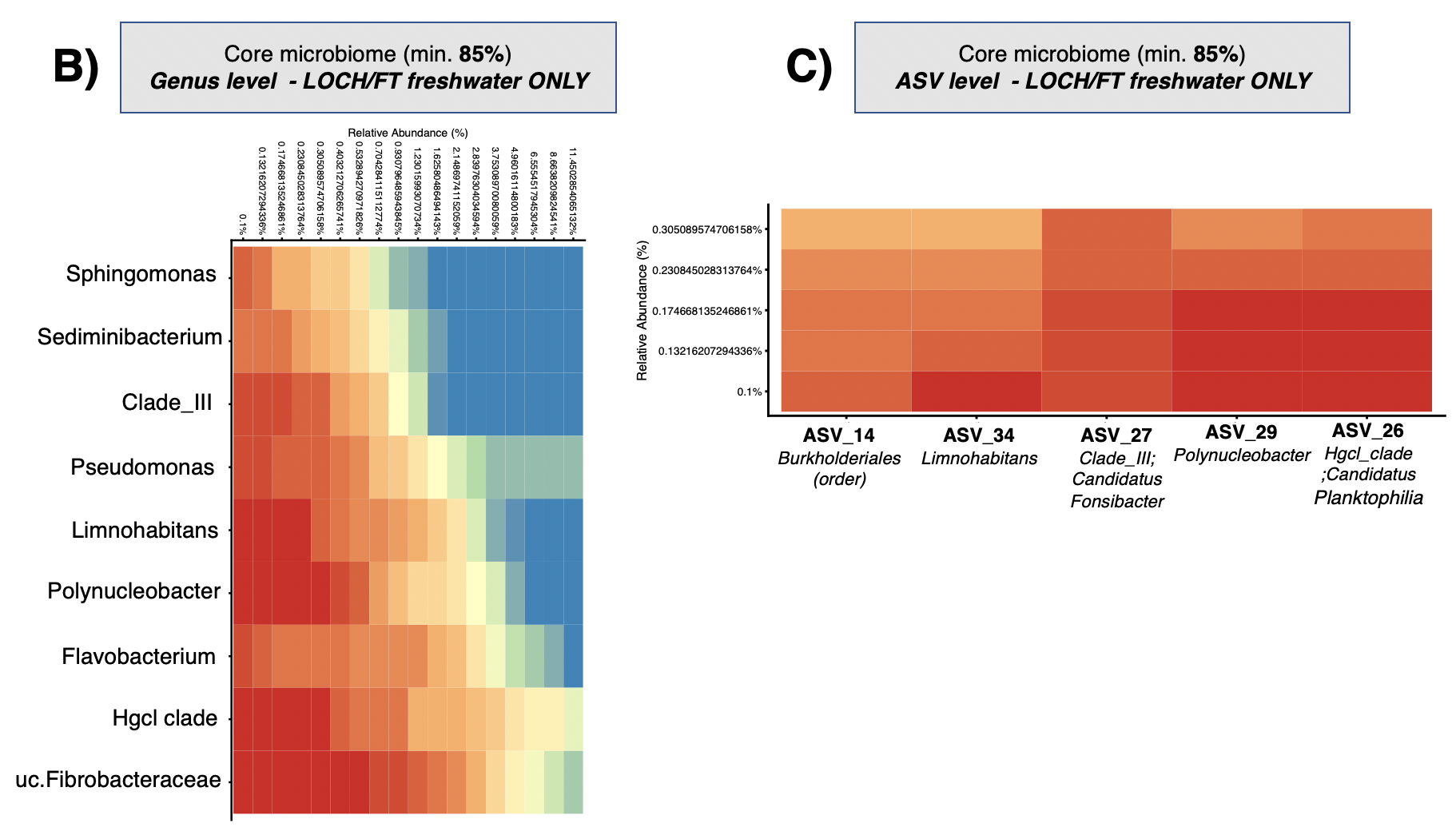


**Supplementary Figure 5. Twenty five most abundant genera of rearing unit surface water for all S1 sampling locations.** Each colour represents an individual genera, apart from yellow which represents other taxa. Each column represents an individual sample, going Glass fibre, Sterivex, Glass fibre, Sterivex for each location.


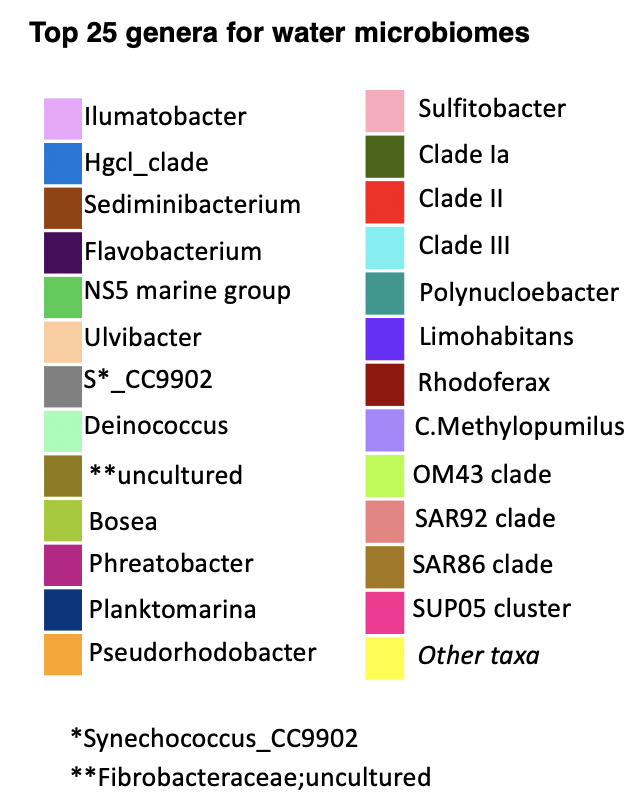

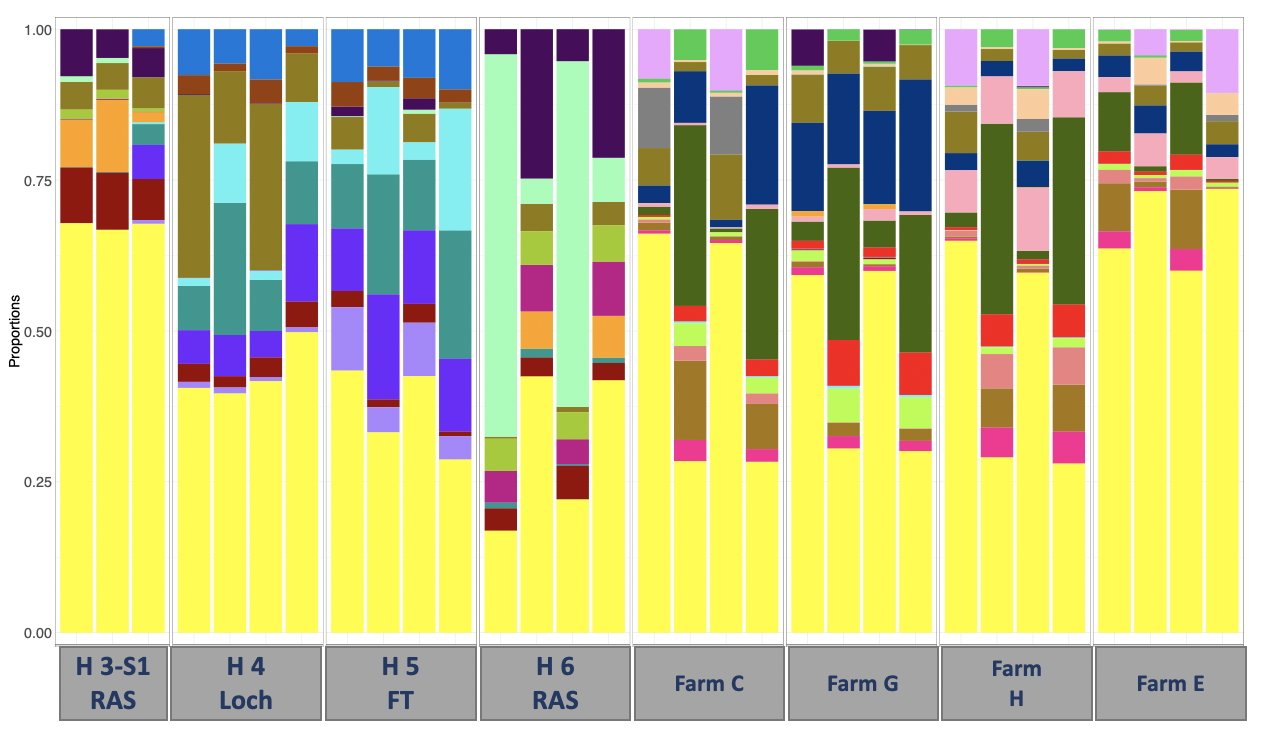

Supplement: Supplementary file 1 — Additional file 1. [file 42523_2024_347_MOESM1_ESM.docx]
